# MiR-93/miR-375: Diagnostic Potential, Aggressiveness Correlation and Common Target Genes in Prostate Cancer

**DOI:** 10.3390/ijms21165667

**Published:** 2020-08-07

**Authors:** Ewa Ciszkowicz, Paweł Porzycki, Małgorzata Semik, Ewa Kaznowska, Mirosław Tyrka

**Affiliations:** 1 Faculty of Chemistry, Rzeszow University of Technology, 35-959 Rzeszów, Poland; semik@prz.edu.pl (M.S.); mtyrka@prz.edu.pl (M.T.); 2 Department of Urology, Municipal Hospital in Rzeszów, 35-241 Rzeszów, Poland; pawel.porzycki@gmail.com; 3 Faculty of Medicine, University of Rzeszów, 35-959 Rzeszów, Poland; e.kaznowska@op.pl

**Keywords:** expression profile, functional annotation, miRNA, prostate cancer, target gene

## Abstract

Dysregulation of miRNAs has a fundamental role in the initiation, development and progression of prostate cancer (PCa). The potential of miRNA in gene therapy and diagnostic applications is well documented. To further improve miRNAs’ ability to distinguish between PCa and benign prostatic hyperplasia (BPH) patients, nine miRNA (-21, -27b, -93, -141, -205, -221, -182, -375 and let-7a) with the highest reported differentiation power were chosen and for the first time used in comparative studies of serum and prostate tissue samples. Spearman correlations and response operating characteristic (ROC) analyses were applied to assess the capability of the miRNAs present in serum to discriminate between PCa and BPH patients. The present study clearly demonstrates that miR-93 and miR-375 could be taken into consideration as single blood-based non-invasive molecules to distinguish PCa from BPH patients. We indicate that these two miRNAs have six common, PCa-related, target genes (*CCND2, MAP3K2, MXI1, PAFAH1B1, YOD1, ZFYVE26*) that share the molecular function of protein binding (GO:0005515 term). A high diagnostic value of the new serum derived miR-182 (AUC = 0.881, 95% confidence interval, CI = 0.816–0.946, *p* < 0.0001, sensitivity and specificity were 85% and 79%, respectively) is also described.

## 1. Introduction

Prostate cancer (PCa) is the most common malignancy among men in Western countries, and a leading cause of male cancer-related deaths [1]. Despite the fact that it tends to grow more slowly than other solid cancers, the PCa diagnosis is challenging due to the heterogeneous nature of the disease, which includes several phenotypes, from indolent to highly aggressive forms [2,3].

Circulating PSA (Prostate-Specific Antigen) is currently the most common non-invasive biomarker used in PCa diagnosis. However, there are many controversies about its use as a screening tool [4] due to the frequently elevated blood levels of PSA among men with benign conditions (e.g., prostatitis, urinary tract infection, or benign prostatic hyperplasia) [5,6]. The screening based on PSA results in a higher incidence of low risk PCa, most of which require no treatment (active surveillance has been proposed to these patients) [4]. The specificity of PSA is poor and its use can lead to over-diagnosis and over-treatment [5,6]. Thus, the invasive procedure of tumour biopsies remains the gold standard for cancer diagnosis [4,7], leading to such complications as bleeding, urinary retention, infection and sepsis [8]. There is a clinical unmet need for predictive, minimal or non-invasive biomarkers to help with optimization of PCa treatment strategies.

MicroRNAs (miRNAs) are a class of non-coding single-stranded RNA molecules containing between 18 and 24 nucleotides that regulate gene expression, both at the transcriptional and post-transcriptional level [9]. A total of 2675 mature human miRNAs with genes located within protein-coding genes or in intergenic regions [10] have already been identified (www.mirbasae.org, 23rd of June 2020). MiRNA targets not only the 3′ untranslated region (3′ UTR) of mRNA, but may also bind to the coding regions or the 5′ untranslated regions (5′ UTRs) [11].

Different multiple miRNAs may interact with a single mRNA and more than one mRNA may be targeted by a single miRNA. Additionally, miRNA can promote or inhibit the expression of many related genes. Thus, large amount of possible miRNA/mRNA interaction may be related with a great number of various miRNAs biological functions, also in tumour development and progression, such as apoptosis, cell cycle regulation, differentiation and mobility [12,13,14,15].

It has been proven that miRNAs are involved in the pathogenesis of various cancers and are considered as potential biomarkers in the cancer detection, including PCa [11,16]. Many independent studies showed that miRNAs may reduce expression of their target tumor suppressor genes and promote oncogenesis, or increase the expression levels of target oncogenes, leading to neoplasia [11,17]. MiRNAs are, therefore, involved in cancer biological behaviours and could be also considered as attractive candidates for cancer therapy [15].

There are several investigations on the mechanisms of oncogenesis and prostate cancer development [18]. Some of them have been well-established, including the androgen receptor mechanism [19,20]; however, the detailed regulations of other mechanisms are still uncertain. In order to improve the diagnosis and management of prostate cancer, the identification of novel effective biomarkers and therapeutic goals is of great importance.

In this study, we aimed to: (a) select and further validate miRNAs with reported high PCa diagnostic capabilities, (b) establish suitability of the selected miRNAs active in the prostate cancer tissue for the serum diagnostic, (c) compare tissue/serum expression profiles of the selected miRNAs set, (d) evaluate the strength of the discriminatory potential of serum-derived miRNAs using receiver-operating characteristic curve (ROC) analysis, and (e) predict target genes and respective biological pathway(s) modulated by the selected tissue-derived miRNAs.

## 2. Results

### 2.1. Patient Characteristics

Patients enrolled in the study were between 48 and 85 years old. No statistically significant differences between the age of PCa patients and control groups were observed. Significant differences in PSA value (ng/mL) were found between BPH and PCa patients, from whom serum samples were taken. The Gleason score (GS) was used for histologic grading. Clinicopathological characteristics of the patient cohorts are summarized in Table 1.

### 2.2. Literature Search

To identify miRNAs with a high discriminatory potential, we set the threshold value of three scientific reports that gave unambiguous results for a given miRNA. Based on comprehensive review of the available literature, nine miRNAs (-21, -27b, -93, -141, -182, -205, -221, -375 and let-7a) meeting this criterion were selected (Table 2). High diagnostic capability and reproducibility of five miRNAs (-21, -93, -141, -375 and let-7a) have already been reported. Four miRNAs (miR-27b, miR-182, miR-221 and miR-205) were included in the study due to extensive results on prostate normal/cancerous tissue [21,22] or cell lines [23,24] and the missing data in the context of serum diagnostic use (Table 2). The diagnostic value of miR-221 and miR-205 in serum was reported in a single reports [25,26].

The Table 2 shows the results of miRNA testing carried out on 1182 serum (842 PCa and 340 non-PCa) and 2876 tissue samples (2058 PCa and 818 non-PCa). For the first time markers miR-27b and miR-182 were evaluated for the liquid (serum) biopsy application combined with analysis of their expression profiles in prostate cancer tissue.

### 2.3. MiRNA Expression in Serum and Tissue

The expression pattern of the nine selected miRNAs was different in 54 tissue specimens (26 PCa, 28 NT samples) and 102 serum samples (40 PCa, 62 BPH). Six out of nine tested miRNAs, miR-21, miR-27b, miR-93, miR-141, miR-182, miR-375, were significantly upregulated in PCa tissue samples. Relative expression ratios of these miRNA were 2.1-, 1.1-, 3.1-, 3.2-, 3.1-, and 3.0-fold higher, respectively (Figure S1). Expressions of miR-205, miR-221 and let-7a in PCa were 0.36-, 0.33 and 0.66-fold reduced in comparison to normal tissue (Figure S1). In serum samples, five different miRNAs (miR-21, miR-93, miR-141, miR-182 and miR-375) were significantly upregulated in PCa patients’ serum with relative expression ratios increased 2.2-, 3.1-, 3.2-, 3.1-, and 2.9-fold, respectively (Figure S2). MiR-205 and let-7a were downregulated (*p* < 0.05) with 0.54- and 0.37-fold decreases of relative expression ratios, respectively (Figure S2). The expression of miR-27b and miR-221 between PCa and BPH serum samples was not significantly different. It is noteworthy that at least two times higher levels of miR-93, miR-141, miR-182 and miR-375 were found both in PCa serum and cancer sections compared to control group.

### 2.4. Correlations of Clinicopathological Variables with miRNAs and Correlations between miRNAs

**Figure 1:**
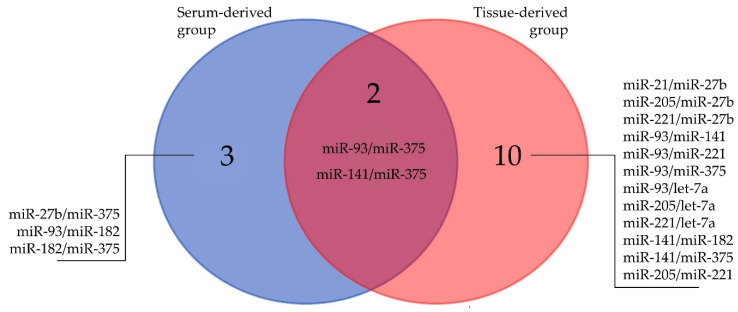
A Venn diagram of the common miRNAs correlations between serum- and tissue-derived groups (for details see Table S2).

Spearman rank (*r_s_*) correlations between clinicopathological variables (age, PSA, Gleason score) and the miRNAs were calculated. No significant correlations were observed between the other clinicopathological factors (PSA, age) and any of the miRNAs (Table S2). The Gleason score was significantly correlated with miRNAs in the serum-derived group: miR-93 (*r_s_* = 0.511, *p* = 0.0007), miR-182 (*r_s_* = 0.460, *p* = 0.0029), miR-375 (*r_s_* = 0.563, *p* = 0.0002), and tissue-derived group: miR-375 (*r_s_* = 0.560, *p* = 0.0029). Statistically significant correlations were observed for pairs miR-93/miR-375 (*r_s_* = 0.678, *p* < 0.0001), miR-27b/miR-375 (*r_s_* = 0.442, *p* = 0.0043), miR-93/miR-182 (*r_s_* = 0.454, *p* = 0.0033), miR-141/miR-375 (*r_s_* = 0.445, *p* = 0.004) and miR-182/miR-375 (*r_s_* = 0.447, *p* = 0.0039) in serum samples. In the group of tissue samples the most significant relationships were indicated for pairs miR-93/miR-141 (*r_s_* = 0.681, *p* = 0.0001), miR-93/miR-375 (*r_s_* = 0.850, *p* < 0.0001), miR-141/miR-375 (*r_s_* = 0.733, *p* < 0.0001) and miR-221/let-7a pairs (*r_s_* = 0.798, *p* < 0.0001) in tissue samples. Common miRNA correlations between serum- and tissue-derived groups are presented on Figure 1. Approximately 8% and 32% of all Spearman rank correlation coefficients were higher than 0.45, while only five and one r_s_ results were over 0.65, respectively, in serum and tissue groups.

Spearman rank (*r*_s_) correlation analysis within the group of PCa patients was performed to evaluate if miRNAs (or miRNAs combinations), separately serum- and tissue-derived, correlate with the clinical aggressiveness of the disease (recommendation for radical prostatectomy (RP)). Based on the European Association of Urology Risk Groups and the latest guidelines on prostate cancer [4] (Table S3), the PCa samples were divided into two subgroups (negative—low-risk EAU group, active surveillance and no recommendation for RP; positive—intermediate- and high-risk EAU group, RP recommendation) [4]. In the group of tissue-derived miRNAs, only miR-375 was significantly correlated with EAU risk groups (*r_s_* = 0.571, *p* = 0.0023), but in the serum-derived group no statistically significant correlation was observed.

### 2.5. Discriminatory Potential of miRNAs

**Figure 2:**
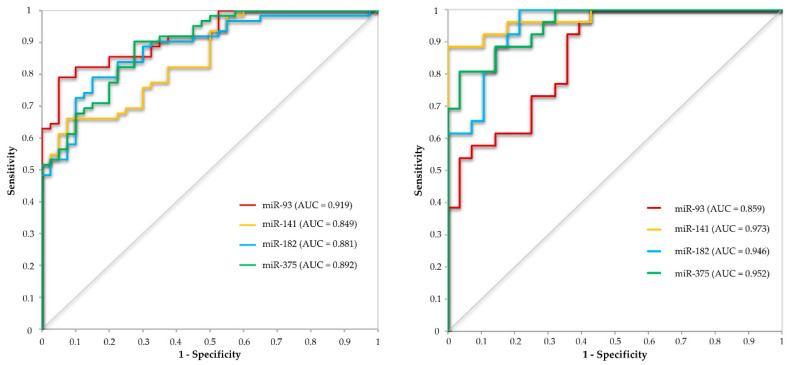
Receiver operating curve (ROC) curve analysis with the use of miRNAs with the highest capability to distinguish between PCa and BPH patients (miR-93, miR-141, miR-182 and miR-375).

Response operating characteristic (ROC) analyses were conducted to evaluate the use of selected miRNAs in distinguishing the PCa from BPH patients and PCa from NT. In the ROC analyses, the optimal cutoff value was chosen to maximize sensitivity and specificity, by applying the Youden’s index (Maximum = Sensitivity + Specificity − 1, Table 3, Figure 2).

The combined ROC analysis of miR-93 and miR-375 was performed in order to evaluate if these miRNAs would supplement each other in a diagnostic test in comparison to single miRNA. The analysis resulted in no further major improvement of the ROC characteristics (sensitivity, specificity, positive predictive value (PPV) or negative predictive value (NPV)). Both in serum and tissue, the combined analysis of miR-93 and miR-375 allowed to obtain intermediate values compared to the ROC values of the particular miRNAs. The exceptions were the sensitivity and the NPV values which decreased in tissue combined ROC analysis (Table 3).

ROC analysis was also performed on the group of PCa samples, both serum and tissue, to evaluate the potential of studied miRNAs in diagnosis of clinically significant PCa with the recommendation for radical prostatectomy (RP). The PCa samples were divided into two subgroups (negative – Gleason score < 7; low-risk EAU group; active surveillance; no recommendation for RP; positive – intermediate (Gleason score 7) and high-risk (Gleason score > 7) EAU group; RP recommendation) based on the European Association of Urology Risk Groups and the latest guidelines on prostate cancer (Table S3) [4].

### 2.6. Prediction of Target Genes and Functional Links between Selected miRNAs and Prostate Cancer

**Figure 3:**
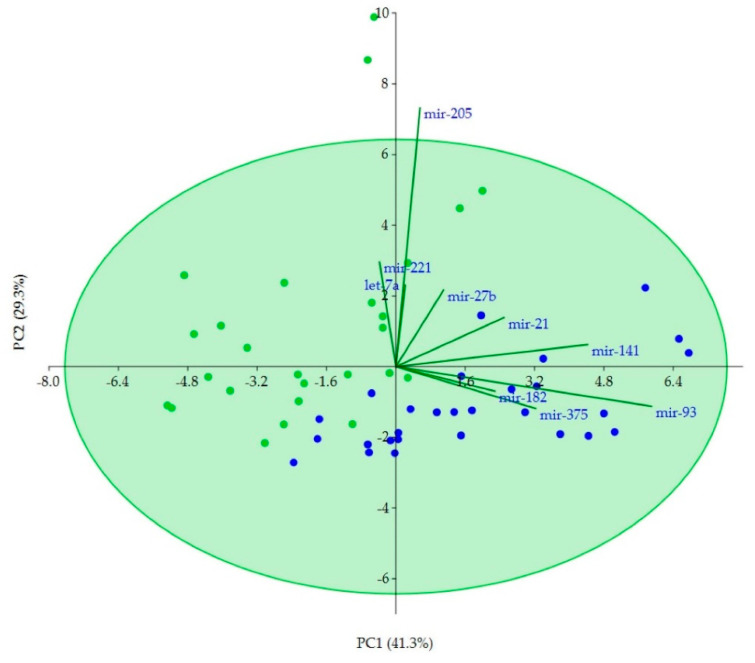
Principal component analysis (PCA) plot on miRNAs expression data from PCa (green dots) and NT (blue dots) tissue samples using the first two components.

The Principal Component Analysis (PCA) showed that miR-93, miR-141 and miR-375 mainly contribute 41.3% of the variation between PCa and NT tissue samples. The second component explains 29.3% of variation associated with expression and is well explained by miR-205 (Figure 3).

A total of 965 miR-93 and/or miR-141 and/or miR-375 separate target genes and 158 miR-205 target genes were selected for functional annotate analysis. A Venn diagram (Figure S2) illustrates the relationships between and among miR-93, miR-141 and miR-375, indicating two common target genes: *CCND2* (cyclin D2) and *PPARA* (peroxisome proliferator-activated receptor alpha). No common genes were found for the set supplemented with 158 targets of miR-205.

**Figure 4:**
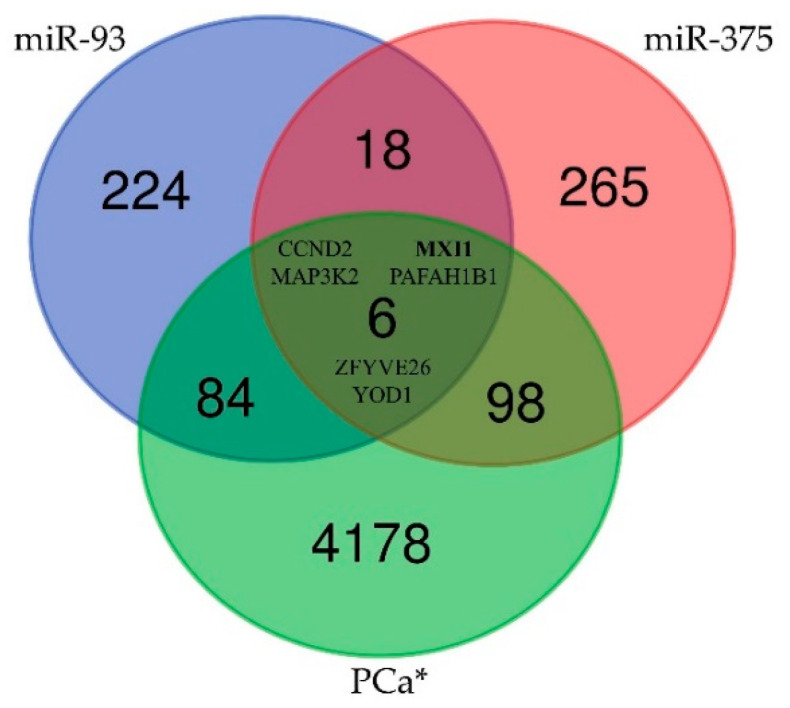
A Venn diagram of the relationships between and among miR-93, miR-375 and genes correlated with PCa indicating six target genes; *: genes in The Human Protein Atlas [64].

**Figure 5:**
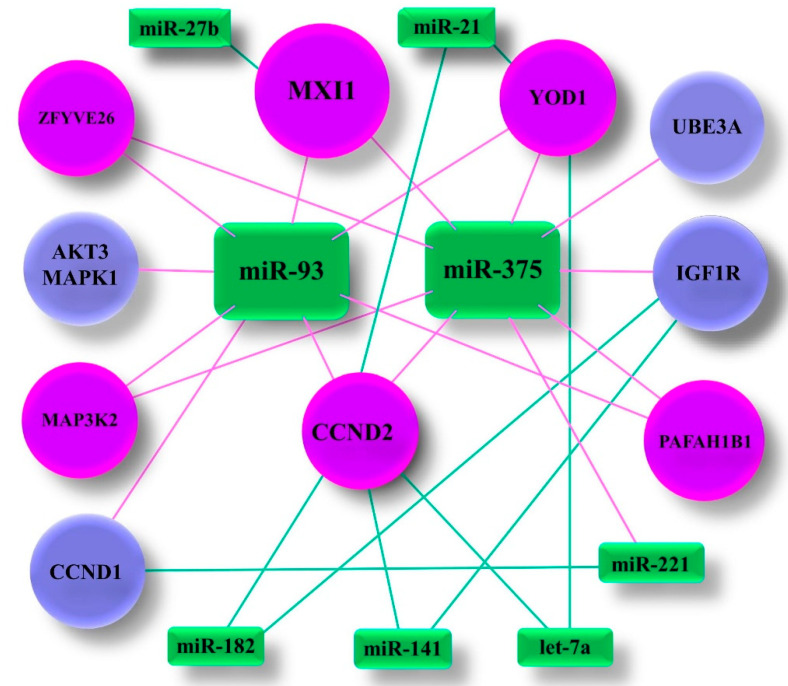
miRNA target gene correlations; *MAPK1* (Mitogen-activated protein kinase 1), *UBE3A* (Ubiquitin protein ligase E3A), *CCND1* (Cyclin D1), *IGF1R* (Insulin like growth factor 1 receptor), *AKT3* (AKT serine/threonine kinase 3).

The Human Protein Atlas database was searched for genes associated with PCa [64]. PPARA was found to be not related with PCa and *CCND2* was identified as PCa unporgnostic with *p* = 0.175. Subsequently, 4366 genes, annotated in the database as PCa prognostic or unprognostic, were included in the comparative analysis to associate PCa related genes with targets for miR-93 and/or miR-141 and/or miR-375. As a result, six PCa related genes, *CCND2, MXI1* (MAX interactor 1), *MAP3K2* (Mitogen-activated protein kinase kinase kinase 2), *PAFAH1B1* (Platelet activating factor acetylhydrolase 1b regulatory subunit 1), *ZFYVE26* (Zinc finger FYVE-type containing 26) and YOD1 (YOD1 deubiquitinase), were identified to be target genes for both miR-93 and miR-375 (Figure 4, Table S4). Additionally, five other genes related with PCa were included in the functional annotation analysis being the common target for miR-93 or miR-375 and one of the other miRNA (miR-21, miR-27b, miR-141, miR-182, miR-221 or let-7a) (Figure 5). As a result a set of 11 target genes were further analysed.

The Database for Annotation, Visualization and Integrated Discovery (DAVID, https://david.ncifcrf.gov/) [65] and DIANA miRpath v.3 database [66] were used to functionally annotate selected genes with 30 Kyoto Encyclopedia of Genes and Genomes (KEGG) terms and 13 Gene Ontology (GO) terms (Table S5).

Interestingly, Prostate cancer, Proteoglycans in cancer and Pathways in cancer signaling were among identified KEEG pathways consisting of target genes connected with miR-93 and/or mir-375, with *p* < 0.0001, *p* = 0.01 and *p* = 0.008, respectively (for details see Table S5).

**Figure 6:**
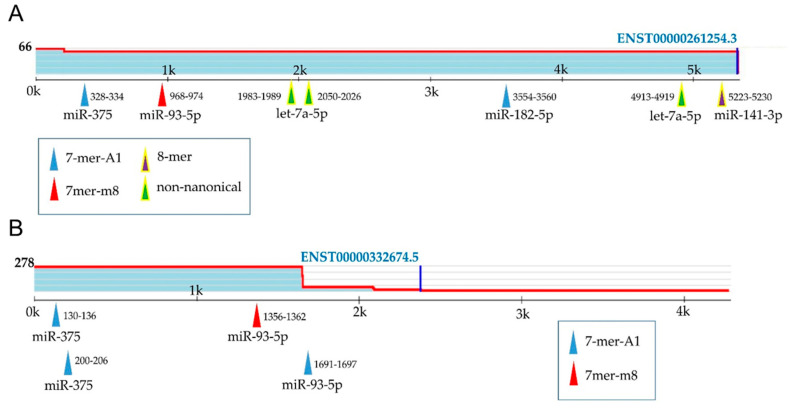
Predicted miRNA binding sites in the human CCND2 3′UTR (NG_034254.1) (**A**) and MXI1 (NG_012103.1) (**B**) were determined using TargetScan.org [67]; 8mer, an exact match to positions 2–8 of the mature miRNA followed by an ‘A’; 7mer-m8, an exact match to positions 2-8 of the mature miRNA; 7mer-A1, an exact match to positions 2–7 of the mature miRNA followed by an ‘A’; non-canonical, sites of other types [67]. Positions in the UTR are written next to the corresponding arrows; sites with the probability of conserved targeting (*P*_CT_) > 0.75 are highlighted with yellow.

According to the public miRNA target binding database (Target Scan 7.2 [67]), binding sites of miR-93 and/or miR-375 are located on CCND2 and MXI1 transcripts (Figure 6A,B). The information about other nine target genes is presented in Table S6.

## 3. Discussion

Since the first identification of miRNAs in prostate cancer cell lines [68] and the first report on the use of biomarkers derived from patients’ serum in the diagnosis of prostate cancer [32], miRNAs have shown great potential as cancer biomarkers. MiRNAs circulating in body fluids, as well as those from prostate tissue, have been consistently described and have broadened the spectrum of miRNAs as potential non-invasive tools for cancer diagnosis [69] and a source of knowledge about the PCa molecular characterization, enabling the indication of target genes regulated by individual miRNAs in the therapeutic context [70].

In our study, differential expression of a set of nine miRNAs was evaluated both in serum and tissue samples of patients with PCa in comparison with BPH and NT samples, respectively. Until now, the expression profiles of miR-182 and miR-27b were described only in PCa tissue [45,48,58], prostate cell lines [21,23], and in liquid biopsy material: whole blood [71] and plasma [70], but not in serum samples. We performed, for the first time, miR-182 and miR-27b expression analysis both on serum (PCa vs. BPH) and tissue samples (PCa vs. NT). Increased expression of miR-182 (3.1-fold change) and no significant differences in miR-27b serum expression levels were found between PCa and BPH patients. MiRNAs expression analysis in serum samples confirmed earlier reports about the overexpression of four miRNAs (miR-21, miR-93, miR-141, and miR-375) and downregulation of two miRNA (miR-205 and let-7a) in PCa serum samples (for References see Table 2). Thus, our results are not in line with reports of let-7a upregulation obtained by Bryant et al. [36], but they confirm the results of Mihelich et al. [30] showing lower let-7a expression level in PCa serum samples in comparison with BPH. Mattie et al. (2006) [72] and Davoren et al. (2008) [73] found let-7a and a combination of let-7a/miR-16, relatively stable as endogenous control genes in prostate cancer tissue. These results were supported by our findings of no statistically significant differences in expression of let-7a in tissue specimens, PCa and NT, which indicates its uncertain relationship with prostate cancer development.

Determination of miRNAs expression levels in tissue samples was in line with the available reports about up-regulation of miR-21, miR-93, miR141, miR-182, miR-375, miR-221 and down-regulation of miR-205, miR-221 and let-7a in PCa tissue samples (for References see Table 2). The results of miR-27b expression profile varies between literature reports. Reduction of miR-27b expression was observed by Goto et al. (2014) [45] and Zhang et al. (2012) [27] between normal PCa and matched PCa tumour tissue samples, however Li et al. [46] described up-regulation of miR-27b in PCa tissue samples in comparison with BPH group. Verdoodt et al. (2013) [58] described lower miR-205 expression between PCa tissue samples with different Gleason scores and miR-205 levels decreased with increasing Gleason scores from 7a = 3 + 4 to 8 = 4 + 4. However, our correlation analysis did not confirm that observation (*p* = 0.1047).

No significant correlations for pairs of clinicopathological characteristics (PSA/age, PSA/Gleason score, age/Gleason score) were observed, which confirms results of many authors [74,75] and indicates the need to look for new biomarkers to facilitate the diagnosis of prostate cancer. Interestingly, only for miR-93, miR-182 and miR-375 associations between expression levels and Gleason score were observed [33,35,76]. This points out the possibility to use of these miRNA markers in prostate cancer prediction studies.

There is a clinical unmet need for predictive, minimally or non-invasive biomarkers to help in the optimization of PCa treatment strategies. In order to distinguish patients who need to have biopsy from those who do not, the information about metrics of ROC analysis are needed. In screening test the predictive values (Positive Predictive Value, PPV, and Negative Predictive Value, NPV) are more informative, however, extremely high values of sensitivity and specificity can be used to make decisions about the individual fate of patients [77]. Due to the lack of definitive answers on the true PSA cut-off point distinguishing cancer from non-cancer, the American Cancer Society Guideline for the Early Detection of Prostate Cancer considers the traditional PSA level of 2.5–4.0 ng/mL as a reasonable threshold for further evaluation. However, a PSA cut-off of 4.0 ng/mL had a sensitivity of 21 percent with the specificity of 91 percent for detection of any prostate cancer, and for detection of a high-grade cancer, the sensitivity was 51 percent [78,79]. However, our research demonstrates even higher sensitivity of the PSA (60%). Surprisingly high diagnostic potential of PSA is observed (AUC = 0.737), however its AUC value was lower than almost all miRNAs tested, except miR-205 and let-7a.

We also showed high discriminatory potential of miR-182 (PCa vs. BPH), but lower than miR-93 and miR-375 (Table 3). Mir-93 was already described as an effective diagnostic and prognostic factor for prostate cancer patients with 100% of sensitivity and specificity [29]. We found that miR-93 is able to distinguish between PCa and BPH patients with 95% sensitivity and 75% PPV, detecting the positive test result and disease presence, respectively. On the other hand, miR-93 shows 79% specificity and 96% NPV detecting negative test result and not present disease. These attributes are also high for miR-141 (Table 3) and confirm the results of Guo et al. (2018) [26] and Porzycki et al. (2018) [28]. Our results together with the results of other authors [26,28,29] indicate the strength of discriminatory potential of these miRNAs. Recently, numerous studies using marker combinations have been conducted [28,53,61] and the discovery of miR-182 as the PCa vs. BPH differentiating factor expands the scope of combinational research with multiple miRNAs.

Nowadays, over-diagnosis and over-treatment are the greatest challenge in the treatment of PCa. Thus, we also attempted to evaluate the potential use of studied miRNAs in distinguishing an aggressive PCa, as diagnosing clinically significant PCa seems to be a significant clinical problem in PCa treatment. Correlation analysis showed relationship between three separate EAU risk groups and miR-375 from tissue samples. Unfortunately, no significant correlation was observed among serum miRNAs. Interestingly, the ROC analysis showed that a single serum miRNA is not capable to discriminate between an aggressive and non-aggressive form of PCa, but such a possibility is provided by the combination of two miRNAs (Table 4). Similar statistically significant AUC values were determined for different pairs of miR-93, miR-182 and miR-375 (AUC between 0.633 and 0.643). The ROC analysis of tissue miRNAs showed that miR-375 could be potentially used in aggressive PCa discrimination with high predictive values and specificity value of 0.92. Additional ROC analysis on different miRNAs combinations did not further improve the diagnostic ability of miR-375. Our results should be confirmed on a larger group of advanced PCa patients and under active surveillance with miRNAs expression levels monitored in time.

Identifying the target genes of the putative miRNAs is important for understanding their role in the aetiology of the disease. In silico analysis by using the miRPath v.3 and DAVID 6.8 platforms allows us to generate a list of target genes and candidate pathways for the differently expressed miRNAs in PCa tissue samples. Stringent target genes selection approach was based on finding a gene with confirmed correlation with particular miRNAs in at least three databases. To discriminate the normal and prostate cancer tissue samples, PCA of the differentially expressed miRNAs was used. This approach reduced the number of the identified miRNAs—target genes by interactions that properly discriminate between patients and healthy controls without losing biological feasibility. In spite of large number of target genes for miR-93, miR-141 and miR-375 (332, 246 and 387 genes, respectively) no common PCa-related target gene was identified [64]. However, significant correlation between expression level of miR-93 and miR-375 was found both in serum and tissue. In consequence, six common genes targeted by miR-93/miR-375 (*CCND2, MAP3K2, MXI1, PAFAH1B1, YOD1, ZFYVE26*) were enrolled in the enrichment analysis (Figure 5). The selection of the remaining five genes (*AKT3, CCND1, IGF1R, MAPK1, UBE3A*) also proved to be correct because all 11 genes were found to enrich the GO:0005515 term–protein binding.

The other, highly statistically significant, enriched GO terms of target genes were protein phosphorylation, cytosol, cell cycle, positive regulation of cyclin-dependent protein serine/threonine kinase activity and positive regulation of cell proliferation. KEGG pathway analysis showed that FoxO signalling, PI3K-Akt signalling, Focal adhesion, Jak-STAT signalling pathways, with a common *CCND2* gene, were also significantly enriched (Table S4). *CCND2*, a crucial cell cycle-regulatory, PCa-related gene, was identified as aberrantly expressed in PCa and many other cancers to regulate cancer cell growth [64,80]. Interestingly, *CCND2* was also identified as the target gene for miR-21, miR-182 (our study), let-7a (this study, [63]) and miR-154 [81], as differentially-expressed miRNA in prostate cancer. Additionally, it has already been proved that the overexpression of *CCND2* inhibited cell growth of prostate cancer, whereas reduced expression promoted cell proliferation of PCa cells and was correlated with tumour progression to high Gleason score and elevated PSA levels [82]. Independent clinical data analysis (18 available clinical data sets, 1095 prostate samples), performed by Chen et al. (2017), highlighted the potential role of *CCND2* in risk stratification and targeted therapy in men with advanced prostate cancer. MXI1, the only prognostic marker among the studied genes [64], is a transcriptional repressor [83] and a tumour suppressor gene, because it acts as an antagonist of the oncogene c-Myc [84], which is overexpressed in many cases of prostate cancer. It was also proven that MXI1 suppresses prostate tumour cell proliferation supporting a role in the pathogenesis of human prostate cancer [85].

Moreover, the *PPAR*α was identified as a target gene for three differentially expressed miRNAs, miR-93, miR-141 and miR-375 (Figure S2), both in tissue and serum samples of PCa patients. PPARα, as well as PPARδ and PPARγ, belong to the nuclear receptor superfamily and can be activated by endogenous or synthetic ligands as transcription factors [86]. *PPAR*δ is the Food and Drug Administration (FDA) approved drug target [64], while *PPAR*γ have been investigated in several clinical trials for their potential in treating non-alcoholic fatty liver disease (NAFLD). Choi et al. (2018) found that NAFLD is associated with the development of PCa [87]. PPARα has already been described as being related with basal phenotype and patient outcome in breast cancer [88], but no relationship with PCa was so far established.

Studies of miRNAs are included in the clinical trials negligible, acting only 0.19% of all prostate cancer clinical trials [89]. At present, recruitment is conducted to nine clinical trials, and only three have been completed so far [89]. This shows the need to work at preclinical stages, i.e., in vitro, in vivo and bioinformatic analysis [90] to gather information about possible clinical applications in the diagnosis and treatment of prostate cancer.

This study preliminarily investigated potential candidate miRNAs involved in the development of PCa, as well as the underlying miRNAs-target genes interactions by in silico analysis, providing potential therapeutic targets for prostate cancer. It is important to further evaluate the correlation between miRNA overexpression and deregulation of six common target genes in prostate cancer and normal cell lines. It will be of great interest to determine whether this signature will prove useful and gain interest in further studies.

## 4. Materials and Methods

### 4.1. Patients Selection and Data Collection

A total of 54 patients submitted to biopsy at the Municipal Hospital in Rzeszow between June 2017 and December 2018 were recruited for our research. Immediately after transrectal ultrasound-guided biopsy (TRUS-biopsy) prostate specimens were formalin-fixed and paraffin-embedded (FFPE specimens). As a result of routine histopathological assessment and systematic sampling for research purposes, 26 cancerous (PCa) and 28 non-cancerous (normal tissue, NT) tissue samples were obtained. Each prostate core biopsy was processed separately, with marking the side from which the biopsy was obtained. The number and length of core was noted. Before embedding, cores were stretched by wrapping them in a piece of paper and coloured with eosin to allow easy visualization during sectioning. Paraffin blocks were cut at 4 µm and at three levels to increase the effectiveness of cancer tissue detection [91]. Histopathologic diagnosis of adenocarcinoma of the prostate was based on light-microscopic examination of basic hematoxylin-eosin–stained tissue sections and immunohistochemistry, if it was necessary. We applied 34bE12/p63/a-methylacyl coenzyme A racemase (AMACR) cocktail immunohistochemistry to identify basal cell keratins and identify cancer. Immunohistochemistry results were evaluated independently by two pathologists. The final report included the histological type of carcinoma, Gleason grading system with Gleason score and Grade group and percentage of tumour involvement per biopsy core. For miRNA analysis 1.5 mm tumour cores were obtained from each paraffin block.

Additionally, 102 serum samples (40 PCa and 62 BPH samples) were collected prospectively from patients with digital rectal examination (DRE) and/or increased prostate-specific antigen (PSA) and patients before TRUS-biopsy. This cohort does not include the patients whose samples were already published in Porzycki et al. [26]. The tissue (*n* = 54) and serum (*n* = 102) cohorts were completely unrelated. The serum collection and haemolysis assessment were conducted as described before [26]. This study was approved by the ethics committee of the Clinical Research Ethics Committee of Subcarpathian Region (“Assessment of the usefulness of miRNAs as biomarkers in diagnosis and prognosis in patients with prostate cancer”, Nr 10/B/2017) with an amendment to the resolution concerning the additional determination of genetic markers from the collected prostate biopsy (Nr 58/B/2017). Written consent was obtained from all patients to provide information and samples for research purposes.

### 4.2. Literature Search

A comprehensive review of the available literature in Web of Science (www.apps.webofknowledge.com) and Scopus (www.scopus.com) databases were performed to identify miRNAs that may contribute to the diagnosis of prostate cancer from patients’ serum. Potentially relevant studies on serum-derived miRNA in PCa were identified using the (a) Scopus string search: (TITLE-ABS-KEY(miRNA) AND TITLE-ABS-KEY (prostate AND cancer) AND TITLE-ABS-KEY (serum); (b) Web of Science string search ALL FIELDS: (miRNA) AND ALL FIELDS: (prostate cancer) AND ALL FIELDS: (serum).

### 4.3. RNA Extraction from Tissue and Serum

RNA from tissue and circulating RNA from serum were obtained using NucleoSpin^®^ total RNA FFPE kit (MACHEREY-NAGEL, Italy) and RiboZolTM RNA Extraction Reagent (AMRESCO LLC, Solon, OH, USA), respectively, according to the manufacturer’s instructions. RNA concentration was measured using Qubit 2.0 fluorometer (INVITROGEN, Carlsbad, CA, USA), and purity was assessed with a JASCO V-670 UV–VIS spectrophotometer (JASCO EUROPE s.r.l., Cremella, Italy). An A260/280 ratio between 1.8 and 1.9 indicated that RNA was free from DNA particles and proteins. Total RNA was divided into aliquots and stored at −80 °C until further use.

### 4.4. cDNA Synthesis

The reverse transcription (RT) reaction mix contained 10 µM of miRNA universal primer (5′-CAG GTC CAG TTT TTT TTT TTT TTT VN-3′) [92], 1 mM each of dNTPs, 1mM ATP, 10 µM of reverse primers specific to reference genes (*RNU6* and *SNORD44*), 10×Poly(A) Pol Reaction Buffer, 200 U of M-MuLV Reverse Transcriptase (200 U/µL, EurX, Gdańsk, Poland), 5 U of Poly(A) polymerase *E. coli* (5000 U/mL, New England Biolabs Inc., UK), and 30 ng of total RNA. RT reaction was carried out at 42 °C/60 min and 95 °C/5 min on Gene Amp^®^ PCR System 9700 (Applied Biosystems, Foster City, CA, USA).

### 4.5. miRNA Expression Analysis

Based on the annotated sequences of mature miRNAs (http://www.mirBase.org) and the selected reference gene (NCBI), specific forward and reverse primers were designed with the use of Primer-BLAST (Table S1) [93]. The comparative analysis of miRNA expression was conducted with GoTaq qPCR Master Mix (Promega, Madison, WI, USA) using the Eco™ Illumina Real-Time PCR System (Illumina, San Diego, CA, USA). Triplicates were performed for each sample and miRNA relative expression levels were calculated by using the comparative Livak 2^−ΔΔCT^ method [94] with RNU6 standing for as the reference gene.

### 4.6. Prediction of the Target Genes of Selected miRNAs

Principal component analysis (PCA) was used to indicate miRNAs with the greatest impact on discrimination between PCa and non-PCa (NT) tissue samples using PAleontological Statistics (PAST) v. 3.25 [95]. PCA analysis was based on differences in the miRNA expression level.

Genes predicted to be targets of the selected miRNAs were considered by Target Scan 7.2 with Conservation (aggregate *P*_CT_ > 0.80, criterion providing low false discovery rates [96]) [68], miRDB with a Target Prediction Score (>80, value chosen to extend the number of predicted target genes, while maintaining a high level of confidence for target prediction [97]) [98], miRpath v.3 (*p*-value < 0.05 statistically significant threshold) [66] and miRWalk (score > 0.80 shows the probability that the results of miRWalk algorithm of miRNA target site prediction are correct [99]) as high-confidence miRNA targets [100]. The predicted targets was selected by at least three databases simultaneously, and then were used for further research. The Database for Annotation, Visualization and Integrated Discovery 6.8 (DAVID, https://david.ncifcrf.gov/) [65] was used to elucidate the molecular functions of the candidate miRNAs.

Target Scan 7.2 was employed to predict effective miRNA target sites in human mRNAs of selected genes. It searches for complementary base pairing between seed region of each miRNA and the 3′UTR heptamer in the target mRNA (conserved 8mer, 7mer and 6mer sites) [67]. The cumulative weighted context++ score (CS) for a specific site is used to calculate the predicted efficacy of targeting [101,102].

### 4.7. Statistical Analysis

Data pre-processing and statistical analyses were performed using GenEx v.6.0.1 (MultiD Analyses AB, Göteborg, Sweden) and STATISTICA v.12 (StatSoft, Tulusa, OK, USA) software. Kruskal–Wallis and Mann–Whitney U non-parametric tests and Spearman rank correlation were used to evaluate differences in miRNAs expression levels and associations between miRNA expression and clinical variables.

Receiver operator characteristics (ROC) curves were constructed to graphically visualize the diagnostic accuracy of each statistically significant miRNA by plotting the sensitivity against the specificity rate. Diagnostic specificity determines the ability to correctly exclude the specific disease and is the ratio of the true negatives to the sum of the true negatives and false positives (Specificity = TP / (TN + FP)). While, diagnostic sensitivity is the ratio of the true positives to the sum of the true positives and the false negatives (Sensitivity = TP/(FN + TP)) and shows the ability to detect patients with the disease. A 1.00 specificity means that all patients without PCa are marked as healthy and a sensitivity of 1.00 means that all patients with PCa are diagnosed, respectively. Positive predictive value (PPV) and negative predictive value (NPV) are the proportion, respectively, of positive results that are true positives and negative results that are true negatives. The area under the curve (AUC) of receiver operated characteristics analysis (ROC) determines the ability of miRNA to distinguish between healthy and PCa patients. An AUC value of 0.5 describes a random effect and a value of 1.0 is the ideal index. Specificity, sensitivity, PPV and NPV were determined for each miRNA by applying cut-off values according to the highest value of Youden’s index obtained in ROC curve analysis [57,70].

To combine miRNAs, logistic regression analysis was applied. Statistical analyses were performed using STATISTICA v.12 (StatSoft, Tulusa OK, USA) software and GenEx v.6.0.1 (MultiD Analyses AB, Göteborg, Sweden). A result was considered statistically significant when *p* < 0.05.

## 5. Conclusions

In conclusion, we demonstrated the high diagnostic capability of severe miRNAs as blood-based non-invasive biomarkers to distinguish PCa from BPH patients. We also compared the expression levels of serum and tissue-derived miRNAs and perform functional analysis for a set of two miRNAs to predict their common target gene/s. According to our knowledge, this is the first attempt to combine differently expressed miRNA target genes to determine their common potential therapeutic goal. Therefore, the molecular mechanism of this key gene in the occurrence of PCa need to be further studied in relation to miRNA deregulation.

## Figures and Tables

**Table 1:** Clinicopathological characteristics of the studied groups.

Characteristics	*Serum Samples*			*p-*Value *^a^*	*Tissue Samples*			*p-*Value ^*a*^
	All	BPH	PCa		All	NT	PCa	
	*n = 102*	*n = 62*	*n = 40*		*n = 54*	*n = 28*	*n = 26*	
**AGE**								
average	67.98	67.93	68.02	0.482	66.90	67.88	65.92	0.716
range	48–85	58–85	48–78		48–79	48–79	48–79	
**PSA** **(ng/mL)**								
average	10.99	7.10	14.88	**0.0001**	12.29	14.47	20.11	0.111
range	2.7–108	2.7–20	3.8–108		3.43–108	3.43–52.6	4.1–108	
2.5–4.0	6	5	1		2	2	0	
4.01–10.0	73	50	23		29	15	14	
10.01–20.0	16	7	9		6	3	3	
>20.0	7	0	7		17	8	9	
**GS 6**			20 (50%)				11 (42.3%)	
**GS 7**			9 (22.5%)				7 (26.9%)	
**GS 8**			5 (12.5%)				3 (11.5%)	
**GS 9**			6 (15%)				5 (19.2%)	
**EAU** **Group Risk**								
Low-risk			15 (37.5%)				13 (50%)	
Intermediate-risk			11 (27.5%)				4 (15.4%)	
High-risk			14 (35%)				9 (34.6%)	

Abbreviations: NT, normal (non-cancerous) tissue; BPH, benign prostatic hyperplasia patients; GS, Gleason score; PSA, prostate specific antigen; EAU, European Association of Urology; ^a^ *p*-values (*Mann-Whitney U test*) indicate significance of the difference between cohorts with and without diagnosed PCa for the clinicopathological variables.

**Table 2:** Differential expression profile of microRNAs in both serum samples and biopsy-derived prostate tissues developed on the basis of comprehensive literature review.

miRNA	Differential Expression in Serum			Differential Expression in Tissue		
**miR-21**	↑	46 PCa/10 BPH	[27]	↑	10 MT/10 AN	[28]
	↑	10 PCa/10 BPH	[25]	↑	53 PCa^c^	[29]
	↑	20 PCa/8 HC	[30]	↑	45 MT/45 AN	[31]
**miR-27b**	**N/R**			↓	49 MT/41 AN	[32]
				↑	63 PCa/28 BPH	[33]
				↓	44 PCa/10 BPH	[34]
**miR-93**	↑	36 PCa/12 HC	[35]	↑	16 MT/16 AN	[36]
	↓	100 PCa/50 BPH	[37]	↑	30 MT/30 BPH	[38]
	↑	112 PCa/48 BPH	[39]			
**miR-141**	↑	25 MetPCa/25 HC	[40]	↑	76 MT/76 AN	[41]
	↑	14 PCa/7 MetPCa 1^a^: 45 PCa, 2^a^: 72 PCa	[42]	↑	36 MT/36 AN	[42]
	↑	21 PCa^b^	[43]	↑	34 PCa/14 BPH	[44]
	↑	36 PCa/12 HC	[35]	↓	55 MT/55 AN	[45]
	↑	25 mCRPC/25 HC	[46]	↑	535 PCa^d^	[1]
	↑	78 PCa/28 HC	[47]	↑	206 MT/29 AN	[48]
	↑	25 mCRPC/25 HC	[49]			
	↑	30 mCRPC/60 locPCa	[50]			
	↑	8 PCa BCR/8 PCa no BCR	[51]			
	↑	31 PCa/13 BPH	[52]			
	↑	72 PCa/34 HC	[26]			
**miR-182**	**N/R**			↑	76 MT/76 AN	[53]
				↑	1^e^: 127 PCa/13 BPH 2^e^: 138 MT/19 AN	[54]
				↑	56 PCa/56 BPH	[22]
				↑	45 MT/45 AN	[31]
**miR-205**	↓	72 PCa/34 HC	[26]	↓	31 MT/31 AN	[55]
				↓	76 MT/76 AN	[53]
				↓	40 MT/40 AN	[56]
				↓	111 MT/111 AN	[57]
				↓	49 PCa/25 BPH	[58]
**miR-221**	↑	10 PCa/10 BPH	[25]	↓	9 PCa/4 BPH	[59]
				↓	76 MT/76 AN	[53]
				↓	40 MT/40 AN	[56]
				↓	45 MT/45 AN	[31]
				↓	206 MT/29 AN	[48]
**miR-375**	↑	14 PCa/7 MetPCa 1^a^: 45 PCa, 2^a^: 72 PCa	[42]	↑	76 MT/76 AN	[53]
	↑	25 mCRPC/25 HC	[46]	↑	76 MT/76 AN	[41]
	↑	78 PCa/28 HC	[47]	↑	1^e^: 127 PCa/13 BPH 2^e^: 138 MT/19 AN	[54]
	↑	25 mCRPC/25 HC	[49]			
	↑	30 mCRPC/60 locPCa	[50]			
	↑	31 PCa/13 BPH	[52]			
	↓	33 PCa/25 BPH	[60]			
**let-7a**	↑	31 PCa/13 BPH	[47]	↓	9 PCa/4 BPH	[59]
	↓	100 PCa/50 BPH	[37]	↓	75 PCa/27 BPH	[61]
				↓	32 PCa BCR/36 PCa no BCR	[62]
				↓	26 MT/26 AN	[63]

Abbreviations: BPH, bengin prostatic hyperplasia patients; HC, healthy control patients; PCa, patients with diagnosed prostate cancer; MT, matched tumor tissue; AN, adjacent normal tissue; locPCa, localized PCa; LocT, localized tumor tissue; MetPCa, patients with metastasized PCa; MetT, metastatic tumour tissue; N/R, no research; PCa BCR, patients with rapid biochemical recurrence; PCa no BCR, patients with no biochemical recurrence; mCRPC, metastatic castration-resistant prostate cancer; 1^a^ and 2^a^ – first and second validation group, respectively, different PCa cancer risk groups; ^b^ – study on therapeutic response in comparison to CTC (Circulating Tumour Cells), LDH (Lactate Dehydrogenase) and PSA; ^c^ – lack of normal prostate tissue samples; ^d^ – study cohort consisted of patients with complete follow-up data; 1^e^ and 2^e^ – training and validation cohort, respectively.

**Table 3:** Discriminatory potential of statistically significant miRNAs and PSA in serum (**A**, *n* = 102) and tissue (**B**, *n* = 54) samples.

**(A) Serum miRNAs**								
**miRNAs**	**AUC**	**95% CI**	SE	***p-Value***	**Sensitivity**	**Specificity**	**PPV**	**NPV**
PSA	0.737	0.636–0.838	0.0517	< 0.0001	0.60	0.79	0.65	0.75
miR-21	0.757	0.661–0.853	0.0490		0.50	0.92	0.80	0.74
miR-93	0.919	0.868–0.934	0.0167		0.95	0.79	0.75	0.96
miR-141	0.849	0.778–0.921	0.0364		0.92	0.66	0.64	0.93
miR-182	0.881	0.816–0.946	0.0331		0.85	0.79	0.72	0.89
miR-205	0.701	0.600–0.801	0.0514		0.90	0.52	0.55	0.89
miR-375	0.892	0.833–0.952	0.0305		0.73	0.90	0.83	0.84
let-7a	0.673	0.564–0.783	0.0561	0.0020	0.40	0.92	0.76	0.70
miR-93/miR-375	0.908	0.870–0.946	0.019	< 0.0001	0.81	0.85	0.78	0.88
**(B) Tissue miRNAs**								
**miRNAs**	**AUC**	**95% CI**	**SE**	***p-Value***	**Sensitivity**	**Specificity**	**PPV**	**NPV**
PSA	0.536	0.380–0.693	0.0800	0.080	0.54	0.54	0.52	0.56
miR-21	0.705	0.562–0.847	0.0726	0.0048	0.36	1.00	1.00	0.64
miR-93	0.859	0.763–0.954	0.0487	<0.0001	0.92	0.64	0.71	0.90
miR-141	0.973	0.935–1.010	0.0193		0.89	1.00	1.00	0.90
miR-182	0.946	0.893–1.000	0.0271		1.00	0.79	0.81	1.00
miR-205	0.688	0.544–0.832	0.0734	0.0104	0.77	0.57	0.63	0.73
miR-221	0.880	0.787–0.974	0.0478	<0.0001	0.77	0.93	0.91	0.81
miR-375	0.952	0.904–1.000	0.0246		0.81	0.96	0.95	0.84
let-7a	0.655	0.504–0.807	0.0772	0.0443	0.39	0.96	0.91	0.63
miR-93/miR-375	0.879	0.817–0.942	0.032	<0.0001	0.69	0.95	0.92	0.77

Abbreviations: AUC, area under the receiver operating characteristics (ROC) curves; CI, confidence interval; SE, standard error; Sensitivity, probability that a test will be positive among diseased patients; Specificity, probability of a negative test result when there is no disease; PPV, positive predictive value, probability of a positive test result when the disease is present; NPV, negative predictive value, probability of s negative test result when the disease is not present.

**Table 4:** miRNAs or combinations of miRNAs from serum (**A**, *n* = 40) and tissue (**B**, *n* = 16) in statistically significant discrimination between aggressive and non-aggressive PCa.

**(A) PCa serum miRNAs**								
**miRNAs**	**AUC**	**95% CI**	**SE**	***p-value***	**Sensitivity**	**Specificity**	**PPV**	**NPV**
miR-21/miR-93	0.640	0.516–0.764	0.063	0.0274	0.50	0.80	0.81	0.49
miR-21/miR-182	0.633	0.508–0.759	0.064	0.0372	0.68	0.60	0.74	0.53
miR-21/miR-375	0.631	0.506–0.756	0.064	0.0403	0.52	0.77	0.79	0.49
miR-93/miR-182	0.643	0.521–0.764	0.062	0.0213	0.70	0.57	0.73	0.53
miR-93/miR-375	0.636	0.514–0.758		0.0283	0.54	0.73	0.77	0.49
miR-182/miR-375	0.641	0.520–0.763		0.0225	0.34	0.93	0.89	0.46
**(B) PCa tissue miRNAs**								
**miRNAs**	**AUC**	**95% CI**	**SE**	***p-value***	**Sensitivity**	**Specificity**	**PPV**	**NPV**
miR-93	0.746	0.542–0.950	0.104	0.0182	0.69	0.69	0.69	0.69
miR-375	0.828	0.653–1.004	0.089	0.0002	0.77	0.92	0.91	0.80
miR-93/miR-141	0.710	0.562–0.858	0.076	0.0055	0.58	0.88	0.83	0.68
miR-93/miR-182	0.692	0.545–0.840	0.075	0.0105	0.50	0.92	0.87	0.65
miR-93/miR-375	0.766	0.635–0.898	0.067	< 0.0001	0.65	0.81	0.77	0.70
miR-141/miR-375	0.743	0.600–0.886	0.073	0.0009	0.58	0.92	0.88	0.69
miR-182/miR-375	0.691	0.542–0.839	0.0757	0.0117	0.46	0.93	0.86	0.63

Abbreviations: AUC, area under the receiver operating characteristics (ROC) curves; CI, confidence interval; SE, standard error; Sensitivity, probability that a test will be positive among diseased patients; Specificity, probability of a negative test result when there is no disease; PPV, positive predictive value, probability of a positive test result when the disease is present; NPV, negative predictive value, probability of s negative test result when the disease is not present.

## Supplementary Materials

The following are available online at https://www.mdpi.com/1422-0067/21/16/5667/s1.

## References

[1] Richardsen E., Andersen S., Melbø-Jørgensen C., Rakaee M., Ness N., Al-Saad S., Nordby Y., Pedersen M.I., Dønnem T., Bremnes R.M. (2019). MicroRNA 141 is associated to outcome and aggressive tumor characteristics in prostate cancer. Sci. Rep..

[2] Ferlay J., Soerjomataram I., Dikshit R., Eser S., Mathers C., Rebelo M., Parkin D.M., Forman D., Bray F. (2015). Cancer incidence and mortality world-wide: Sources, methods and major patterns in GLOBOCAN 2012. Int. J. Cancer.

[3] Siegel R.L., Miller K.D., Jemal A. (2018). Cancer statistics. CA Cancer J. Clin..

[4] Mottet N., Cornford P., Van den Bergh R.C.N., Briers E., De Santis M., Fanti S., Gillessen S., Grummet J., Henry A.M., Lam T.B. (2020). EAU-EANM-ESTRO-ESUR-SIOG Guidelines on prostate cancer. Eur. Assoc. Urol..

[5] Adhyam M., Gupta A.K. (2012). A review on the clinical utility of PSA. Indian J. Surg. Oncol..

[6] Bokhorst L.P., Valdagni R., Rannikko A., Kakehi Y., Pickles T., Bangma C.H., Roobol M.J. (2016). PRIAS study group. A decade of active surveillance in the PRIAS study: An update and evaluation of the criteria used to recommend a switch to active treatment. Eur. Urol..

[7] Zedan A.H., Hansen T.F., Assenholt J., Pleckaitis M., Madsen J.S., Osther P.J.S. (2018). microRNA expression in tumour tissue and plasma in patients with newly diagnosed metastatic prostate cancer. Tumour Biol..

[8] Takudome S., Ando R., Koda Y. (2016). Discoveries and application of prostate-specific antigen, and some proposals to optimize prostate cancer screening. Cancer Manag. Res..

[9] Catalanotto C., Cogoni C., Zardo G. (2016). MicroRNA in control of gene expression: An overview of nuclear functions. Int. J. Mol. Sci..

[10] Zedan A.H., Hansen T.F., Assenholt J., Madsen J.S., Osther P.J.S. (2019). Circulating miRNAs in localized/locally advanced prostate cancer patients after radical prostatectomy and radiotherapy. Prostate.

[11] Chen C.Z. (2005). MicroRNAs as Oncogenes and Tumor Suppressors. N. Engl. J. Med..

[12] Xu P., Vernooy S.Y., Guo M., Hay B.A. (2003). The drosophila microRNA Mir-14 suppresses cell death and is required for normal fat metabolism. Curr. Biol..

[13] Croce C.M. (2009). Causes and consequences of microRNA dysregulation in cancer. Nat. Rev. Genet..

[14] Mens M.M.J., Ghanbari M. (2018). Cell cycle regulation of stem cells by microRNAs. Stem Cell Rev..

[15] Ji W., Sun B., Su C. (2017). Targeting MicroRNAs in Cancer Gene Therapy. Genes.

[16] Sita-Lumsden A., Dart D.A., Waxman J., Bevan C.L. (2013). Circulating microRNAs as potential new biomarkers for prostate cancer. Br. J. Cancer.

[17] Xu W., Liu M., Peng X., Zhou P., Zhou J., Xu K., Xu H., Jiang S. (2013). miR-24-3p and miR-27a-3p promote cell proliferation in glioma cells via cooperative regulation of MXI1. Int. J. Oncol.

[18] Wei J., Yin Y., Deng Q., Zhou J., Wang Y., Yin G., Yang J., Tang Y. (2020). Integrative Analysis of MicroRNA and Gene Interactions for Revealing Candidate Signatures in Prostate Cancer. Front. Genet..

[19] Filella X., Foj L. (2017). MiRNAs as a novel biomarkers in the management of prostate cancer. Clin. Chem. Lab. Med..

[20] Liu R.S.C., Olkhov-Mitsel E., Jeyapala R., Zhao F., Commisso K., Klotz L., Loblaw A., Liu S.K., Vesprini D., Fleshner N.E. (2018). Assessment of serum microRNA biomarkers to predict reclassification of prostate cancer in patients on active surveillance. J. Urol..

[21] Wang D., Lu G., Shao Y., Xu D. (2018). MiR-182 promotes prostate cancer progression through activating Wnt/β-catenin signal pathway. Biomed. Pharmacother..

[22] Baumann B., Acosta A.M., Richards Z., Deaton R., Sapatynska A., Murphy A., Kajdacsy-Balla A., Gann P.H., Nonn L. (2019). Association of high miR-182 levels with low-risk prostate cancer. Am. J. Pathol..

[23] Gandellini P., Giannoni E., Casamichele A., Taddei M.L., Callari M., Piovan C., Valdagni R., Pierotti M.A., Zaffaroni N., Chiarugi P. (2014). miR-205 hinders the malignant interplay between prostate cancer cells and associated fibroblasts. Antioxid. Redox Signal..

[24] Ippolito L., Marini A., Cavallini L., Morandi A., Pietrovito L., Pintus G., Giannoni E., Schrader T., Puhr M., Chiarugi P. (2016). Metabolic shift toward oxidative phosphorylation in docetaxel resistant prostate cancer cells. Oncotarget.

[25] Kotb S., Mosharafa A., Essawi M., Hassan H., Meshref A., Morsy A. (2014). Circulating miRNAs 21 and 221 as biomarkers for early diagnosis of prostate cancer. Tumour Biol..

[26] Guo X., Han T., Hu P., Guo X., Zhu C., Wang Y., Chang S. (2018). Five microRNAs in serum potential biomarkers for prostate risk assessment and therapeutic intervention. Int. Urol. Nephrol..

[27] Zhang H.L., Yang L.F., Zhu Y., Yao X.D., Zhang S.L., Dai B., Zhu Y.P., Shen Y.J., Shi G.H., Ye D.W. (2011). Serum miRNA-21: Elevated levels in patients with metastatic hormone-refractory prostate cancer and potential predictive factor for the efficacy of docetaxel-based chemotherapy. Prostate.

[28] Melbø-Jørgensen C., Ness N., Andersen S., Valkov A., Dønnem T., Al-Saad S., Kiselev Y., Berg T., Nordby Y., Bremnes R.M. (2014). Stromal expression of miR-21 predicts biochemical failure in prostate cancer patients with Gleason score 6. PLoS ONE.

[29] Leite K.R.M., Reis S.T., Viana N., Morais D.R., Moura C.M., Silva I.A., Pontes J., Katz B., Srougi M. (2015). Controlling RECK miR-21 promotes tumor cell invasion and is related to biochemical recurrence in prostate cancer. J. Cancer.

[30] Porzycki P., Ciszkowicz E., Semik M., Tyrka M. (2018). Combination of three miRNA (miR-141, miR-21, and miR-375) as potential diagnostic tool for prostate cancer recognition. Int. Urol. Nephrol..

[31] Kurul N.O., Ates F., Yilmaz I., Narli G., Yesildal C., Senkul T. (2019). The association of let-7c, miR-21, miR-145, miR-182, and miR-221 with clinicopathologic parameters of prostate cancer in patients diagnosed with low-risk disease. Prostate.

[32] Goto Y., Kojima S., Nishikawa R., Enokida H., Chiyomaru T., Kinoshita T., Nakagawa M., Naya Y., Ichikawa T., Seki N. (2014). The microRNA-23b/27b/24-1 cluster is a disease progression marker and tumor suppressor in prostate cancer. Oncotarget.

[33] Li T., Sun X., Liu Y. (2017). miR-27b expression in diagnosis and evaluation prognosis of prostate cancer. Int. J. Clin. Exp. Pathol..

[34] Pimenta R.C.A., Viana N.I., Amaral G.Q., Park R., Morais D.R., Pontes J., Guimaraes V.R., Camargo J.A., Leite K.R.M., Nahas W.C. (2018). MicroRNA-23b and microRNA-27b plus flutamide treatment enhances apoptosis rate and decreases CCNG1 expression in a castration -resistant prostate cancer cell line. Tumor Biol..

[35] Moltzahn F., Olshen A.B., Baehner L., Peek A., Fong L., Stöppler H., Simko J., Hilton J.F., Carroll P., Blelloch R. (2011). Microfluidic-based multiplex qRT-PCR identifies diagnostic and prognostic microRNA signatures in the sera of prostate cancer patients. Cancer Res..

[36] Liu M.X., Liao J., Xie M., Gao Z.K., Wang X.H., Zhang Y., Shang M.H., Yin L.H., Pu Y.P., Liu R. (2018). Mir-93-5p transferred by exosomes promotes the proliferation of esophageal cancer cells via intercellular communication by targeting PTEN. Biomed. Environ. Sci..

[37] Mihelich B.L., Maranville J.C., Nolley R., Peehl D.M., Nonn L. (2015). Elevated serum microRNA levels associate with absence of high-grade prostate cancer in a retrospective cohort. PLoS ONE.

[38] Yang K., Li Y.W., Gao Z.Y., Xiao W., Li T.Q., Song W., Zheng J., Chen H., Chen G.H., Zou H.Y. (2019). MiR-93 functions as a tumor promoter in prostate cancer by targeting disabled homolog 2 (DAB2) and an antitumor polysaccharide from green tea (*Camellia sinensis*) on their expression. Int. J. Biol. Makromol..

[39] Gao Y., Deng K., Liu X., Dai M., Chen X., Chen J., Chen J., Huang Y., Dai S., Chen J. (2019). Molecular mechanism and role of microRNA-93 inhuman cancers: A study based on bioinformatics analysis, meta-analysis, and quantitative polymerase chain reaction validation. J. Cell Biochem..

[40] Mitchell P.S., Parkin R.K., Kroh E.M., Fritz B.R., Wyman S.K., Pogosowa-Agadjanyan R.L., Peterson E., Noteboom J., O’Briant K.C., Allen A. (2008). Circulating microRNAs as Stable Blood-Based Markers for Cancer Detection. Proc. Natl. Acad. Sci. USA.

[41] Wach S., Nolte E., Szczyrba J., Stöhr R., Hartmann A., Ømtoft T., Dyrskjøt L., Eltze E., Wieland W., Keck B. (2012). MicroRNA profiles of prostate carcinoma detected by multiplatform microRNA screening. Int. J. Cancer.

[42] Brase J.C., Johannes M., Schlomm T., Falth M., Haese A., Steuber T., Beissbarth T., Kuner R., Sültmann R. (2011). Circulating miRNAs are correlated with tumor progression in prostate cancer. Int. J. Cancer.

[43] Gonzales J.C., Fink L.M., Goodman O.B., Symanowski J.T., Vogelzang N.J., Ward D.C. (2011). Comparison of circulating microRNA 141 to circulating tumor cells, lactate dehydrogenase, and prostate-specific antigen for determining treatment response in patients with metastatic prostate cancer. Clin. Genitourin. Cancer.

[44] Paziewska A., Mikula M., Dabrowska M., Kulecka M., Goryca K., Antoniewicz A., Dobruch J., Borowka A., Rutkowski P., Ostrowski J. (2018). Candidate diagnostic miRNAs that can detect cancer in prostate biopsy. Prostate.

[45] Xu S., Ge J., Zhang Z., Zhou W. (2018). miR-141 inhibits prostatic cancer cell proliferation and migration, and induces cell apoptosis via targeting of RUNX1. Oncol. Rep..

[46] Selth L.A., Townley S., Gillis J.L., Ochnik A.M., Murti K., Macfarlane R.J., Chi K.N., Marshall V.R., Tilley W.D., Butler L.M. (2012). Discovery of circulating microRNAs associated with human prostate cancer using a mouse model of disease. Int. J. Cancer.

[47] Bryant R.J., Pawlowski T., Catto J.W.F., Marsden G., Vessella R.L., Rhees B., Kuslich C., Visakorpi T., Hamdy F.C. (2012). Changes in circulating microRNA levels associated with prostate cancer. Br. J. Cancer.

[48] Zhao Z., Weickmann S., Jung M., Lein M., Kilic E., Stephan C., Erbersdobler A., Fendler A., Jung K. (2019). A novel predictor tool of biochemical recurrence after radical prostatectomy based on a five-microRNA tissue signature. Cancers.

[49] Cheng H.H., Mitchell P.S., Kroh E.M., Dowell A.E., Chéry L., Siddiqui J., Nelson P.S., Vessella R.L., Knudsen B.S., Chinnaiyan A.M. (2013). Circulating microRNA profiling identifies a subset metastatic prostate cancer patients with evidence cancer-associated hypoxia. PLoS ONE.

[50] Nguyen P.V., Srihari S., Leong H.W. (2013). Identifying conserved protein complexes between species by constructing interolog networks. BMC Bioinform..

[51] Selth L.A., Townley S.L., Bert A.G., Stricker P.D., Sutherland P.D., Horwath L.G., Goodall G.J., Butler L.M., Tilley W.D. (2013). Circulating microRNAs predict biochemical recurrence in prostate cancer patients. Br. J. Cancer.

[52] Haldrup C., Kosaka N., Ochiya T., Borre M., Høyer S., Orntoft T.F., Sorensen K.D. (2014). Profiling of circulating microRNAs for prostate cancer biomarker discovery. Drug Deliv. Transl. Res..

[53] Schaefer M., Pollex T., Hanna K., Tuorto F., Meusburger M., Helm M., Lyko F. (2010). RNA methylation by Dnmt2 protects transfer RNAs against stress-induced cleavage. Genes Dev..

[54] Kristensen H., Thomsen A.R., Haldrup C., Dyrskjøt L., Høyer S., Borre M., Mouritzen P., Ørntoft T.F., Sørensen K.D. (2016). Novel diagnostic and prognostic classifiers for prostate cancer identified by genome-wide microRNA profiling. Oncotarget.

[55] Gandellini P., Folini M., Longoni N., Pennati M., Binda M., Colecchia M., Salvioni R., Supino R., Moretti R., Limonta P. (2009). miR-205 exerts tumor-suppressive functions in human prostate through down-regulation of protein kinase Cε. Cancer Res..

[56] Srivastava A., Goldberger H., Dimtchev A., Ramalinga M., Chijioke J., Marian C., Oermann E.K., Uhm S., Kim J.S., Chen L.N. (2013). MicroRNA profiling in prostate cancer-the diagnostic potential of urinary miR-205 and miR-214. PLoS ONE.

[57] Verdoodt B., Neid M., Vogt M., Kuhn V., Liffers S.T., Palisaar R.J., Noldus J., Tannapfel A., Mirmohammadsadegh A. (2013). MicroRNA-205, a novel regulator of the anti-apoptotic protein Bcl2, is downregulated in prostate cancer. Int. J. Oncol.

[58] Hagman Z., Haflidadóttir B., Ceder J., Larne O., Bjartell A., Lilja H., Edsjö A., Ceder Y. (2013). miR-205 negatively regulates the androgen receptor and is associated with adverse outcome of prostate cancer patients. Br. J. Cancer.

[59] Porkka K.P., Pfeiffer M.J., Waltering K.K., Vessella R.L., Tammela T.L., Visakorpi T. (2007). MicroRNA expression profiling in prostate cancer. Cancer Res..

[60] Dülgeroğlu Y., Eroğlu O. (2018). Diagnostic performance of microRNAs in the circulation in differential diagnosis of BPH, chronic prostatitis and prostate cancer. Turk. J. Biochem..

[61] Kelly B.D., Miller N., Sweeney K.J., Durkan G.C., Rogers E., Walsh K., Kerin M.J. (2015). A circulating microRNA signature as a biomarker for prostate cancer in a high risk group. J. Clin. Med..

[62] Tian B., Huo N., Li Y., He Z. (2015). let-7a and its target, insulin-like growth factor 1 receptor, are differentially expressed in recurrent prostate cancer. Int. J. Mol. Med..

[63] Dong Q., Meng P., Wang T., Qin W., Qin W., Wang F., Yuan J., Chen Z., Yang A., Wang H. (2010). MicroRNA Let-7a Inhibits Proliferation of Human Prostate Cancer Cells In Vitro and In Vivo by Targeting E2F2 and CCND2. PLoS ONE.

[64] The Human Protein Atlas. https://www.proteinatlas.org/.

[65] Dennis G., Sherman B.T., Hosack D.A., Yang J., Gao W., Lane H.C., Lempicki R.A. (2003). DAVID: Database for annotation, visualization, and integrated discovery. Genome Biol..

[66] Vlachos I.S., Zagganas K., Paraskevopoulou M.D., Georgakilas G., Karagkouni D., Vergoulis T., Dalamagas T., Hatzigeorgiou A.G. (2015). DIANA-miRPath v3.0: Decipherng microRNA function with experimental support. Nucleic Acids Res..

[67] TargetScanHuman. http://www.targetscan.org/vert_72/.

[68] Jiang J., Lee E.J., Gusev Y., Schmittgen T.D. (2005). Real-time expression profiling of microRNA precursors in human cancer cell lines. Nucleic Acids Res..

[69] Bidarra D., Constâncio V., Barros-Silva D., Ramalho-Carvalho J., Moreira-Barbosa C., Antunes L., Maurício J., Oliveira J., Henrique R., Jerónimo C. (2019). Circulating microRNAs as biomarkers for prostate cancer detection and metastasis development prediction. Front. Oncol..

[70] Lewis B.P., Shih I.H., Jones-Rhoades M.W., Bartel D.P., Burge C.B. (2003). Prediction of mammalian microRNA targets. Cell.

[71] Bryzgunova O.E., Lekchnov E.A., Zaripov M.M., Yurchenko Y.B., Yarmoschuk S.V., Pashkovskaya O.A., Rykova E.Y., Zheravin A.A., Laktionov P.P. (2017). Bioinformatics analysis for evaluation of the diagnostic potentialities of miR-19b, -125b and-205 as liquid biopsy markers of prostate cancer. AIP Conf. Proc..

[72] Mattie M.D., Benz C.C., Bowers J., Sensinger K., Wong L., Scott G.K., Fedele V., Ginzinger D., Getts R., Haqq C. (2006). Optimized high-throughput microRNA expression profiling provides novel biomarker assessment of clinical prostate and breast cancer biopsies. Mol. Cancer.

[73] Davoren P.A., McNeill R.E., Lowery A.J., Kerin M.J., Miller N. (2008). Identification of suitable endogenous control genes for microRNA gene expression analysis in human breast cancer. BMC Mol. Biol..

[74] Aghdam S.G., Ebrazeh M., Hemmatzadeh M., Seyfizadeh N., Shabgah A.G., Azizi G., Ebrahimi N., Babaie F., Mohammadi H. (2019). The role of microRNAs in prostate cancer migration, invasion, and metastasis. J. Cell Physiol..

[75] Daniyal M., Siddiqui Z.A., Akram M., Asif H.M., Sultana S., Khan A. (2014). Epidemiology, etiology, diagnosis and treatment of prostate cancer. Asian Pac. J. Cancer Prev..

[76] Kachakova D., Mitkova A., Popov E., Popov I., Vlahova A., Dikov T., Christova S., Mitev V., Slavov C., Kaneva R. (2015). Combinations of serum prostate-specific antigen and plasma expression levels of let-7c, miR-30c, miR-141, and miR-375 as potential better diagnostic biomarkers for prostate cancer. DNA Cell Biol..

[77] Trevethan R. (2017). Sensitivity, specificity, and predictive values: Foundations, pliabilities, and pitfalls in research and practice. Front. Public Health.

[78] Wolf A.M.D., Wender R.C., Etzioni R.B., Thompson I.M., D’Amico A.V., Volk R.J., Brooks D.D., Dash C., Guessous I., Andrews K. (2010). American Cancer Society guideline for the early detection of prostate cancer. CA Cancer J. Clin..

[79] American Cancer Society American Cancer Society Recommendations for Prostate Cancer Early Detection. https://www.cancer.org/cancer/prostate-cancer/detection-diagnosis-staging/acs-recommendations.html.

[80] Sun Y., Jia X., Hou L., Liu X. (2016). Screening of differently expressed miRNA and mRNA in prostate cancer by integrated analysis of transcription data. Urology.

[81] Zhu C., Shao P., Bao M., Li P., Zhou H., Cai H., Cao Q., Tao L., Meng X., Ju X. (2014). miR-154 inhibits prostate cancer cell proliferation by targeting CCND2. Urol. Oncol..

[82] Chen Y., Zhang Q., Wang Q., Li J., Sipeky C., Xia J., Gao P., Hu Y., Zhang H., Yang X. (2017). Genetic association analysis of the RTK/ERK pathway with aggressive prostate cancer highlights the potential role of CCND2 in disease progression. Sci. Rep..

[83] UniProt UniProtKB-P50539 (MXI1_HUMAN). https://www.uniprot.org/uniprot/P50539.

[84] Zhou J., Wang W., Gao Z., Peng X., Chen X., Chen W., Xu W., Xu H., Lin M.C., Jiang S. (2013). MicroRNA-155 Promotes Glioma Cell Proliferation via the Regulation of MXI1. PLoS ONE.

[85] Taj M.M., Tawil R.J., Engstrom L.D., Zeng Z., Hwang C., Sanda M.G., Wechsler D.S. (2001). Mxi1, a Myc Antagonist, Suppresses Proliferation of DU145 Human Prostate Cells. Prostate.

[86] Wahli W., Martinez E. (1991). Superfamily of steroid nuclear receptors: Positive and negative regulators of gene expression. FASEB J..

[87] Choi Y.J., Lee D.H., Han K.D., Yoon H., Shin C.M., Park Y.S., Kim N. (2018). Is nonalcoholic fatty liver disease associated with the development of prostate cancer? A nationwide study with 10,516,985 Korean men. PLoS ONE.

[88] Baker B.G., Ball G.R., Rakha E.A., Nolan C.C., Caldas C., Ellis I.O., Green A.R. (2013). Lack of expression of the proteins GMPR2 and PPARα are associated with the basal phenotype and patient outcome in breast cancer. Breast Cancer Res. Treat..

[89] US National Library of Medicine Studies for: Prostate Cancer. https://clinicaltrials.gov/ct2/results?cond=Prostate+Cancer&term=miRNA&cntry=&state=&city=&dist=.

[90] Hanna J., Hossain G.S., Kocerha J. (2019). The Potential for microRNA Therapeutics and Clinical Research. Front. Genet..

[91] Van der Kwast T., Bubendorf L., Mazeroles C., Raspollini M.R., Van Leenders G.J., Pihl C.-G., Kujala P. (2013). Guidelines on processing and reporting of prostate biopsies: The 2013 update of the Pathology Committee of the European Randomized Study of Screening for Prostate Cancer (ERSPC). Virchows Arch..

[92] Alvarez M.L., Doné S.C., Alvarez M., Nourbakhsh M. SYBR_®_ Green and TaqMan_®_ Quantitative PCR Arrays: Expression Profile Genes Relevant to a Pathway or a Disease State. RNA Mapping.

[93] Ye J., Coulouris G., Zaretskaya I., Cutcutache I., Rozen S., Madden T. (2012). Primer-BLAST: A tool to design target-specific primers for polymerase chain reaction. BMC Bioinform..

[94] Livak K.J., Schmittgen T.D. (2001). Analysis of relative gene expression data using real-time quantitative PCR and the 2^−∆∆CT^ method. Methods.

[95] Hammer Ø., Harper D.A.T., Ryan P.D. (2001). Past: Paleontological Statistics Software Package for education and data analysis. Palaeo Electron..

[96] Lu J., Clark A.G. (2012). Impact of microRNA regulation on variation in human gene expression. Genome Res..

[97] Wang X. (2008). miRDB: A microRNA target prediction and functional annotation database with a wiki interface. RNA.

[98] Chen Y., Wang X. (2020). miRDB: An online database for prediction of functional microRNA targets. Nucleic Acids Res..

[99] Dweep H., Gretz N. (2015). miRWalk2.0: A comprehensive atlas of microRNA-target interactions. Nat. Methods.

[100] Baek D., Villén J., Shin C., Camargo F.D., Gygi S.P., Bartel D.P. (2008). The impact of microRNAs on protein output. Nature.

[101] Lewis B.P., Burge C.B., Bartel D.P. (2005). Conserved seed pairing, often flanked by adenosines, indicates that thousands of human genes are microRNA targets. Cell.

[102] Agarwal V., Bell G.W., Nam J.W., Bartel D.P. (2015). Predicting effective microRNA target sites in mammalian mRNAs. Elife.

