# Supplementary material for: MiR-93/miR-375: Diagnostic Potential, Aggressiveness Correlation and Common Target Genes in Prostate Cancer
Source: Int J Mol Sci. 2020 Aug 7;21(16):5667. doi: 10.3390/ijms21165667 (PMC7460886; doi:10.3390/ijms21165667)
Supplement: Supplementary file 1 [file ijms-21-05667-s001.pdf]

Table S1. MicroRNAs and reference genes used for real-time qPCR analyses.

miRNA / reference gene name	miRBase ID[a] / NCBI ID[b]	miRBase[a] / NCBI Accession No.[b]	Sequence<br>Primer Forward (5′ – 3′)<br>Primer Reverse (5′ – 3′)	Chromosome Location	miRNA Gene Family
**miRNAs**					
**miR-21**	**hsa-miR-21-5p**	0000076	UAGCUUAUCAGACUGAUGUUGA<br>5′ - GCAGTAGCTTATCAGACTGATG - 3′<br>5′ - GGTCCAGTTTTTTTTTTTTTTTCAAC - 3′	chr17: 59841266-59841337 [+]	miR-21
**miR-27b**	**hsa-miR-27b-5p**	0004588	AGAGCUUAGCUGAUUGGUGAAC<br>5′ - GAGAGCTTAGCTGATTGGTG - 3′<br>5′ - GGTCCAGTTTTTTTTTTTTTTTGTTC - 3′	chr9: 95085445-95085541 [+]	miR-27
**miR-93**	**hsa-miR-93-5p**	0000093	CAAAGUGCUGUUCGUGCAGGUAG<br>5′ - GCAAAGTGCTGTTCGTG - 3′<br>5′ - GTCCAGTTTTTTTTTTTTTTTCTACCT - 3′	chr7: 100093768-100093847 [-]	miR-17
**miR-141**	**hsa-miR-141-3p**	0000432	UAACACUGUCUGGUAAAGAUGG<br>5′ - CGCAGTAACACTGTCTGGT - 3′<br>5′ - GTCCAGTTTTTTTTTTTTTTTCCATCT - 3′	chr12: 6964097-6964191 [+]	miR-8
**miR-182**	**hsa-miR-182-5p**	0000259	UUUGGCAAUGGUAGAACUCACACU<br>5′ - GTTTGGCAATGGTAGAACTCA - 3′<br>5′ - GGTCCAGTTTTTTTTTTTTTTTAGTGT - 3′	chr7: 129770383-129770492 [-]	miR-182
**miR-205**	**hsa-miR-205-5p**	0000266	UCCUUCAUUCCACCGGAGUCUG<br>5′ - CCTTCATTCCACCGGAGT - 3′<br>5′ - GGTCCAGTTTTTTTTTTTTTTTCAGA - 3′	chr1: 209432133-209432242 [+]	miR-205
**miR-221**	**hsa-miR-221-5p**	0004568	ACCUGGCAUACAAUGUAGAUUU<br>5′ - GCAGACCTGGCATACAATG - 3′<br>5′ - GGTCCAGTTTTTTTTTTTTTTTAAATCTAC - 3′	chrX: 45746157-45746266 [-]	miR-221
**miR-375**	**hsa-miR-375**	0000728	GCGACGAGCCCCUCGCACAAACC<br>5′ - AGTTTGTTCGTTCGGCTC - 3′<br>5′ - GGTCCAGTTTTTTTTTTTTTTTCAC - 3′	chr2: 219001645-219001708 [-]	miR-375
**let-7a**	**hsa- let-7a-1**	0000062	UGAGGUAGUAGGUUGUAUAGUU<br>5′ - GCAGTGAGGTAGTAGGTTG - 3′<br>5′ - GGTCCAGTTTTTTTTTTTTTTTAACTATAC - 3′	chr9: 94175957-94176036 [+]	let-7

**Reference gene**				
**RNU6**	**RNU6 (SNORD6. U6)**	004394[b]	GTGCTCGCTTCGGCAGCACATATACTAAAATTGGAACGAT ACAGAGAAGATTAGCATGGCCCCTGCGCAAGGATGACAC GCAAATTCGTGAAGCGTTCCATATTTT<br>5′ - CGCTTCGGCAGCACATATAC - 3′<br>5′ - AGGGGCCATGCTAATCTTCT - 3′	15q23

[a] – Sanger Institute. Manchester. UK; http://www.mirbase.org
[b] - National Center of Biotechnology Information (NCBI) Reference Sequence Number; https://www.ncbi.nlm.nih.gov/nucleotide/

Table S2. Spearman rank correlation coefficients (r_s) between clinicopathological factors and the examined microRNAs in all tissues (n = 54) and serum (n = 102) samples. The significantly differentially expressed miRNAs and correlations are highlighted in **bold** and indicated as: *** $p < 0.0001$, ** $p < 0.0005$, * $p < 0.005$); the correlation strength is marked with an appropriate color, the darker the color, the greater the relationship between variables.

		**Serum-derived miRNAs**											
	r_s	**Age**	**GS**	**PSA(ng /mL)**	**miR-21**	**miR-27b**	**miR-93**	**miR-141**	**miR-182**	**miR-205**	**miR-221**	**miR-375**	**let-7a**
**Tissue-derived miRNAs**	**Age**	-	0.267	0.174	–0.045	–0.247	0.035	0.171	0.202	0.034	0.022	0.221	0.106
	**GS**	0.101	-	0.421	0.157	0.054	**0.511****	0.299	**0.460***	0.053	0.344	**0.563****	0.276
	**PSA (ng/mL)**	0.341	0.094	-	0.124	–0.276	0.172	0.146	0.042	0.131	–0.059	–0.022	–0.212
	**miR-21**	0.276	–0.306	0.172	-	0.161	0.388	0.107	0.354	–0.023	–0.050	0.256	0.069
	**miR-27b**	0.505	–0.277	0.416	**0.602***	-	0.310	0.181	0.322	0.328	0.111	**0.442***	0.360
	**miR-93**	0.342	0.401	0.174	0.271	0.436	-	0.383	**0.454***	0.035	0.211	**0.678*****	0.201
	**miR-141**	0.156	0.176	0.485	0.333	0.331	**0.681****	-	0.229	0.005	0.032	**0.445***	0.100
	**miR-182**	0.081	0.120	0.524	0.217	0.363	0.407	**0.643****	-	0.159	–0.015	**0.447***	0.275
	**miR-205**	0.234	–0.344	0.283	0.249	**0.586***	0.182	0.255	0.099	-	–0.016	0.091	0.037
	**miR-221**	0.329	–0.117	0.195	0.238	**0.652****	**0.644****	0.441	0.451	**0.635****	-	0.435	–0.056
	**miR-375**	0.314	**0.560***	0.359	0.249	0.372	**0.850*****	**0.733*****	0.465	0.169	0.456	-	0.089
	**let-7a**	0.134	–0.140	0.140	0.240	0.424	**0.569***	0.470	0.466	**0.574***	**0.798*****	0.409	-

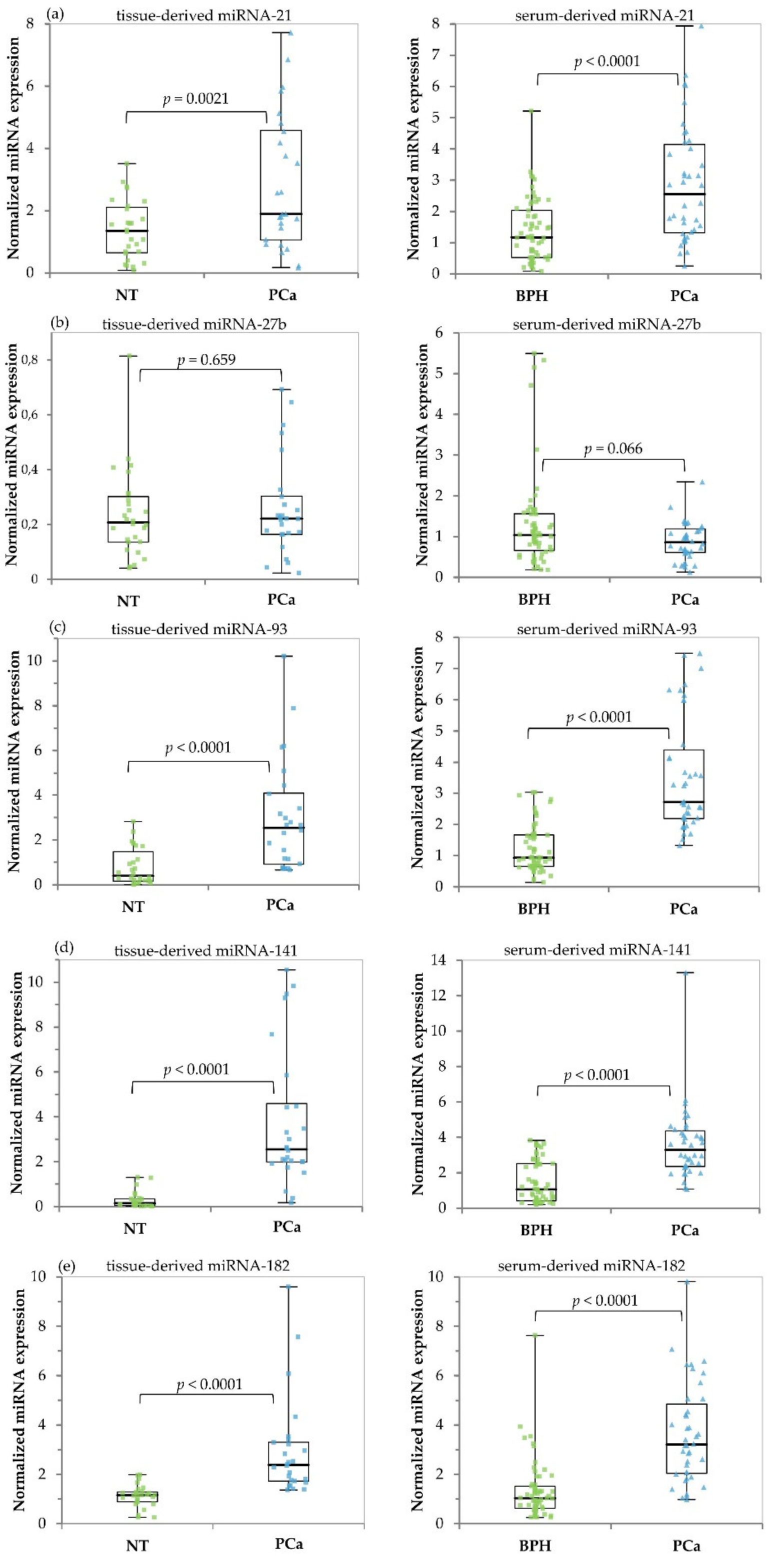

Figure S1. Expression of nine miRNAs (a – i) analyzed in tissue- (NT = 28, PCa = 26) and serum-derived (BPH = 62, PCa = 40) samples. Normalized miRNA expression data are given as box- and whisker plots. Boxes represent the lower and upper quartiles with medians, while whiskers illustrate the ranges of the miRNAs expression levels.

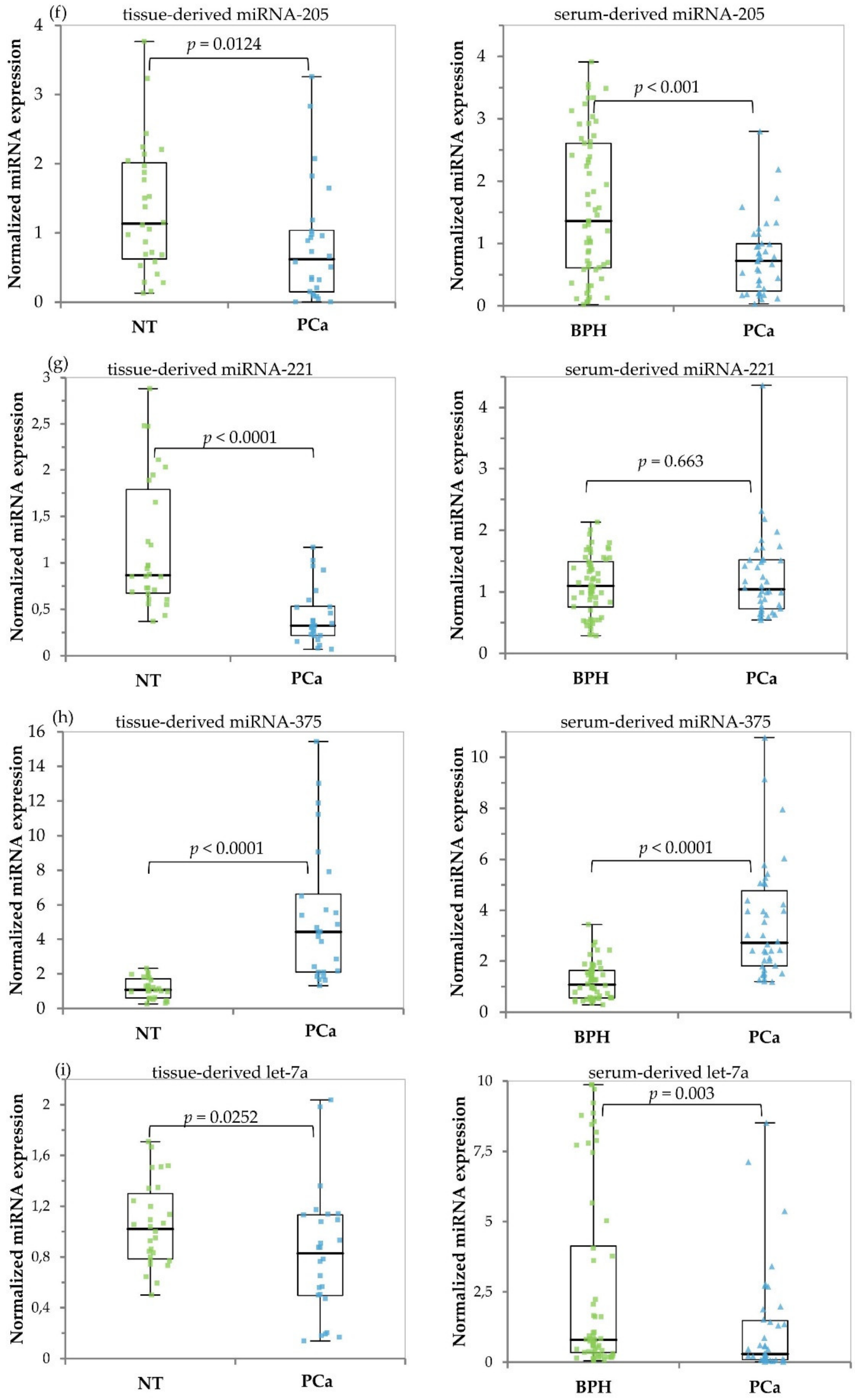

Figure S1 continued. Expression of nine miRNAs (a–i) analyzed in tissue- (NT = 28, PCa = 26) and serum-derived (BPH = 62, PCa = 40) samples. Normalized miRNA expression data are given as box- and whisker plots. Boxes represent the lower and upper quartiles with medians, while whiskers illustrate the ranges of the miRNAs expression levels.

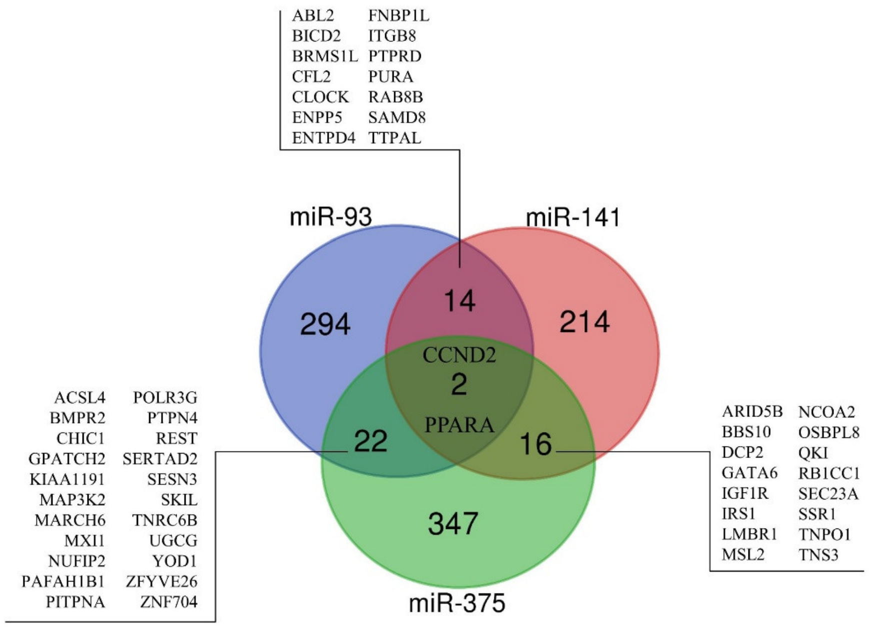

Figure S2. A Venn diagram of the relationships between and among miR-93, miR-141, and miR-375, indicating two common target genes (*CCND2* and *PPARA*).

Table S3. Target genes for miR-93 and/or miR-375 described in The Human Protein Atlas database as PCa prognostic and unprognostic [64].

Ensemble	Gene	Gene description	Chromosome (position)	Pathology prognostics	*p* value
ENSG00000117020	AKT3	AKT serine/threonine kinase 3	1 (243488233–243851079)	Unprognostic	0.137
ENSG00000110092	CCND1	Cyclin D1	11 (69641087–69654474)	Unprognostic	0.044
ENSG00000118971	CCND2	Cyclin D2	12 (4273772 –4305350)	Unprognostic	0.175
ENSG00000140443	IGF1R	Insulin like growth factor 1 receptor	15 (98648971–98964530)	Unprognostic	0.162
ENSG00000169967	MAP3K2	Mitogen-activated protein kinase kinase kinase 2	2 (127298730–127388465)	Unprognostic	0.139
ENSG00000100030	MAPK1	Mitogen-activated protein kinase 1	22 (21754500–21867680)	Unprognostic	0.104
ENSG00000119950	MXI1	MAX interactor 1, dimerization protein	10 (110207605–110287365)	Prognostic unfavourable	**<0.0001**
ENSG00000007168	PAFAH1B1	Platelet activating factor acetylhydrolase 1b regulatory subunit 1	17 (2593210–2685615)	Unprognostic	0.058

ENSG00000114062	UBE3A	Ubiquitin protein ligase E3A	15 (25333728-25439056)	Unprognostic	0.048
ENSG00000180667	YOD1	YOD1 deubiquitinase	1 (207043849-207052980)	Unprognostic	0.010
ENSG00000072121	ZFYVE26	Zinc finger FYVE-type containing 26	14 (67727374-67816590)	Unprognostic	0.037

Table S4. GO terms and KEGG pathways for miR-93 and miR-375 PCa-related target genes [64] based on in silico through DAVID [65] and DIANA miRpath v.3 database [66].

No.	KEGG pathway Category	Genes	*p* value
1	hsa04068:FoxO signaling pathway	MAPK1, IGF1R, CCND1, CCND2, AKT3	<0.0001
2	hsa04151:PI3K-Akt signaling pathway		<0.0001
3	hsa04510:Focal adhesion		<0.0001
4	hsa04917:Prolactin signaling pathway	MAPK1, CCND1, CCND2, AKT3	<0.0001
5	hsa05200:Pathways in cancer	MAPK1, IGF1R, CCND1, AKT3	0.008
6	hsa05203:Viral carcinogenesis	MAPK1, CCND1, CCND2, UBE3A	0.001
7	hsa05205:Proteoglycans in cancer	MAPK1, IGF1R, CCND1, AKT3	0.001
8	hsa05214:Glioma		<0.0001
9	hsa05215:Prostate cancer		<0.0001
10	hsa05218:Melanoma		<0.0001
11	hsa04010:MAPK signaling pathway	MAPK1, MAP3K2, AKT3	0.033
12	hsa04014:Ras signaling pathway	MAPK1, IGF1R, AKT3	0.026
13	hsa04015:Rap1 signaling pathway		0.023
14	hsa04066:HIF-1 signaling pathway		0.005
15	hsa04152:AMPK signaling pathway	IGF1R, CCND1, AKT3	0.008
16	hsa04550:Signaling pathways regulating pluripotency of stem cells	MAPK1, IGF1R, AKT3	0.011
17	hsa04630:Jak-STAT signaling pathway	CCND1, CCND2, AKT3	0.011
18	hsa04914:Progesterone-mediated oocyte maturation	MAPK1, IGF1R, AKT3	0.004
19	hsa04919:Thyroid hormone signaling pathway	MAPK1, CCND1, AKT3	0.007
20	hsa05161:Hepatitis B		0.011
21	hsa05162:Measles	CCND1, CCND2, AKT3	0.010
22	hsa05166:HTLV-I infection		0.033
23	hsa05210:Colorectal cancer	MAPK1, CCND1, AKT3	0.002
24	hsa05212:Pancreatic cancer		0.002
25	hsa05213:Endometrial cancer		0.002
26	hsa05220:Chronic myeloid leukemia		0.003
27	hsa05221:Acute myeloid leukemia		0.002
28	hsa05223:Non-small cell lung cancer		0.002
29	hsa05216:Thyroid cancer	MAPK1, CCND1	0.033
30	hsa05219:Bladder cancer		0.047

No.	Category	GO term	Genes	*p* value
1	BP	GO:0006468~protein phosphorylation	MAPK1, CCND1, MAP3K2, AKT3	0.002
2	BP	GO:0007049~cell cycle	MAPK1, CCND1, CCND2	0.007
3	BP	GO:0008284~positive regulation of cell proliferation	MAPK1, IGF1R, CCND2	0.030
4	BP	GO:0030968~endoplasmic reticulum unfolded protein response	CCND1, YOD1	0.026
5	BP	GO:0033598~mammary gland epithelial cell proliferation	MAPK1, CCND1	0.005
6	BP	GO:0043627~response to estrogen		0.038
7	BP	GO:0045737~positive regulation of cyclin-dependent protein serine/threonine kinase activity	CCND1, CCND2	0.014
8	CC	GO:0005829~cytosol	MAPK1, CCND1, CCND2, UBE3A, MAP3K2, PAFAH1B1, YOD1	0.004
9	CC	GO:0000307~cyclin-dependent protein kinase holoenzyme complex	CCND1, CCND2	0.008

10	MF	GO:0005515~protein binding	MAPK1, IGF1R, CCND1, CCND2, UBE3A, MAP3K2, ZFYVE26, PAFAH1B1, YOD1, MXI1, AKT3	0.001
11	MF	GO:0004672~protein kinase activity	CCND1, MAP3K2, AKT3	0.018
12	MF	GO:0004674~protein serine/threonine kinase activity	MAPK1, MAP3K2, AKT3	0.020
13	MF	GO:0019901~protein kinase binding	CCND1, CCND2, MAP3K2	0.020

Abbreviations: KEGG, Kyoto Encyclopedia of Genes and Genomes; GO, Gene Ontology; BP, biological processes; CC, cell component; MF, molecular function.

Table S5. miRNAs targets among 11 analyzed genes predicted by TargetScan [67].

Gene	miRNA	Position in the UTR	Seed match	CS	P_{ct}
**AKT3** ENST00000366539.1 3' UTR length: 5530	miR-93	1142–1148	7mer-m8	–0.16	0.74
**CCND1** ENST00000227507.2 3' UTR length: 3192	miR-93	583–589	7mer-1A	–0.03	0.2
		1014–1020	7mer-m8	–0.21	0.74
**CCND2** ENST00000261254.3 3' UTR length: 5347	miR-93	968–974	7mer-m8	–0.2	0.74
	miR-375	328–334	7mer-1A	–0.08	0.35
**IGF1R** ENST00000268035.6 3' UTR length: 7088	miR-93	3998–4004	7mer-1A	–0.01	<0.1
	miR-375	2276–2282	7mer-1A	–0.01	0.29
		2993–2999	7mer-1A	–0.01	<0.1
**MAP3K2** ENST00000409947.1 3' UTR length: 8910	miR-93	65–71	7mer-m8	–0.18	0.74
		265–272	8mer	–0.18	0.96
		6553–6559	7mer-m8	–0.2	0.68
		8869–8875	7mer-m8	–0.14	0.74
		1503-1509	7mer-1A	–0.01	<0.1
		3342–3348	7mer-m8	–0.02	<0.1
		5996–6002	7mer-1A	–0.01	0.17
	miR-375	2760–2766	7mer-1A	–0.01	0.22
		1552–1558	7mer-1A	–0.01	<0.1
**MAPK1** ENST00000215832.6 3' UTR length: 9750	miR-93	445–452	8mer	–0.23	0.96
		414–420	7mer-1A	–0.05	0.13
		7597–7603	7mer-m8	–0.02	<0.1
**MXI1** ENST00000332674.5 3' UTR length: 4283	miR-93	1356–1362	7mer-m8	–0.15	0.74
		1691–1697	7mer-1A	–0.01	0.27
		2830–2836	7mer-m8	–0.02	<0.1
	miR-375	130–136	7mer-1A	–0.13	0.35
		200–206	7mer-1A	–0.16	0.35
**PAFAH1B1** ENST00000397195.5 3' UTR length: 3813	miR-93	2870–2877	8mer	–0.16	0.89
	miR-375	1249–1255	7mer-1A	–0.05	<0.1
**PPAR**α ENST00000396000.2 3' UTR length: 8376	miR-93	416–422	7mer-m8	–0,15	<0.1
		97–103	7mer-1A	–0,01	<0.1
		620–626	7mer-m8	–0,02	<0.1
		1969–1975	7mer-m8	–0,02	<0.1
		4966–4972	7mer-m8	–0,02	<0.1
	miR-375	3040–3046	7mer-1A	–0,07	<0.1
**UBE3A** ENST00000232165.3 3' UTR length: 1960	miR-93	1637–1643	7mer-m8	–0.07	<0.1
	miR-375	218–224	7mer-1A	–0.16	0.35
**YOD1** ENST00000315927.4 3′ UTR length: 5182	miR-93	1138–1144	7mer-m8	–0.13	0.2
		3495–3502	8mer	–0.24	0.94
		1601–1607	7mer-m8	–0.09	<0.1
		2217–2223	7mer-1A	–0.03	0.17
	miR-375	85–91	7mer-1A	–0.11	0.31
**ZFYVE26** ENST00000347230.4 3'UTR length: 1916	miR-93	353–360	8mer	–0.2	0.91
		1659–1666	8mer	–0.23	0.94
	miR-375	321–327	7mer-1A	–0.14	0.35

Abbreviations: CS, the context++ score for a specific site is the sum of the contribution of 14 features [100]; P_{CT}, probability of conserved targeting; 3′ UTR, untranslated region; 8mer, an exact match to positions 2–8 of the mature miRNA (the seed + position 8) followed by an 'A'; 7mer-m8, an exact match to positions 2–8 of the mature miRNA (the seed + position 8); 7mer-A1, an exact match to positions 2–7 of the mature miRNA (the seed) followed by an 'A' [67].
